# A Novel Metagenomic Short-Chain Dehydrogenase/Reductase Attenuates *Pseudomonas aeruginosa* Biofilm Formation and Virulence on *Caenorhabditis elegans*

**DOI:** 10.1371/journal.pone.0026278

**Published:** 2011-10-26

**Authors:** Patrick Bijtenhoorn, Hubert Mayerhofer, Jochen Müller-Dieckmann, Christian Utpatel, Christina Schipper, Claudia Hornung, Matthias Szesny, Stephanie Grond, Andrea Thürmer, Elzbieta Brzuszkiewicz, Rolf Daniel, Katja Dierking, Hinrich Schulenburg, Wolfgang R. Streit

**Affiliations:** 1 Abteilung für Mikrobiologie und Biotechnologie, Biozentrum Klein Flottbek, Universität Hamburg, Hamburg, Germany; 2 EMBL Hamburg Outstation, Hamburg, Germany; 3 Institut für Organische Chemie, Eberhard Karls Universität Tübingen, Tübingen, Germany; 4 Laboratorium für Genomanalyse, Institut für Mikrobiologie und Genetik, Georg-August-Universität Göttingen, Göttingen, Germany; 5 Department of Evolutionary Ecology and Genetics, Christian-Albrechts Universität zu Kiel, Kiel, Germany; Consejo Superior de Investigaciones Cientificas, Spain

## Abstract

In *Pseudomonas aeruginosa*, the expression of a number of virulence factors, as well as biofilm formation, are controlled by quorum sensing (QS). *N*-Acylhomoserine lactones (AHLs) are an important class of signaling molecules involved in bacterial QS and in many pathogenic bacteria infection and host colonization are AHL-dependent. The AHL signaling molecules are subject to inactivation mainly by hydrolases (Enzyme Commission class number EC 3) (i.e. *N*-acyl-homoserine lactonases and *N*-acyl-homoserine-lactone acylases). Only little is known on quorum quenching mechanisms of oxidoreductases (EC 1). Here we report on the identification and structural characterization of the first NADP-dependent short-chain dehydrogenase/reductase (SDR) involved in inactivation of *N*-(3-oxo-dodecanoyl)-L-homoserine lactone (3-oxo-C_12_-HSL) and derived from a metagenome library. The corresponding gene was isolated from a soil metagenome and designated *bpiB09*. Heterologous expression and crystallographic studies established BpiB09 as an NADP-dependent reductase. Although AHLs are probably not the native substrate of this metagenome-derived enzyme, its expression in *P. aeruginosa* PAO1 resulted in significantly reduced pyocyanin production, decreased motility, poor biofilm formation and absent paralysis of *Caenorhabditis elegans*. Furthermore, a genome-wide transcriptome study suggested that the level of *lasI* and *rhlI* transcription together with 36 well known QS regulated genes was significantly (≥10-fold) affected in *P. aeruginosa* strains expressing the *bpiB09* gene in pBBR1MCS-5. Thus AHL oxidoreductases could be considered as potent tools for the development of quorum quenching strategies.

## Introduction

Autoinducers play important roles as quorum-sensing signals in bacterial cell-cell communication (Quorum sensing) [1]. Through the accumulation of bacterially produced signaling molecules the bacterial population is able to sense increases in cell density and alter gene expression accordingly [2]. This allows coordinated expression of genes at the population level involved in behaviors, which are most effective at higher cell densities, such as pathogenicity, biofilm formation, production of extracellular proteins and others. For an excellent review on interspecies signaling, we refer to Shank and Kolter [3]. Many quorum sensing mechanisms in Gram-negative bacteria involve N-AHLs, signal molecules whose general mechanism of synthesis is well understood.

By now, about 20 proteins belonging to 10 different clusters have been found interfering with these bacterial quorum sensing molecules. Most of these enzymes belong to the enzyme class of hydrolases. Mainly two types of hydrolases act on autoinducer I molecules: (i) The majority of the identified enzymes are lactonases (EC 3.1.1.–) and this group of enzymes has been reviewed recently [4]. More recently three additional lactonases have been published [5]–[7]. Lactonases hydrolyze the lactone ring in a reversible way. (ii) Furthermore acylases (EC 3.5.1.–) are known to interfere with the autoinducer I-like molecules. Aminoacylases cleave the lacton ring off the fatty acids. Acylases have been identified in a variety of microorganisms: *Comamonas* sp. [8], *P. aeruginosa* [9], [10], *P. syringae* [11], *Ochrobactrum* sp. [12], *Ralstonia* sp. [13], *Rhodococcus erythropolis* [14], *Shewanella* sp. [15] and *Streptomyces sp.* [16].

Surprisingly, only very few oxidoreductases (EC 1) have been found to influence quorum sensing controlled phenotypes. Up to date only one, a P-450/NADPH-P450 monooxygenase (UniProt P14779) has been isolated from *Bacillus megaterium* and characterized in detail [17], [18]. The respective AHL oxidizing enzyme was designated CYP102A1 and it was able to oxidize both long chained AHLs and fatty acids with varying chain length at various positions. The two different domains would catalyze reactions classified as EC 1.14.14.1 and EC 1.6.2.4. In addition Uroz and colleagues reported the presence of oxidoreductase activities in crude cell extracts of *Rhodococcus erythropolis* W2 [14]. They demonstrated that crude cell extracts of *R. erythropolis* reduced the 3-oxo-substituent of 3-oxo-C_14_-HSL to yield the corresponding 3-hydroxy derivative, 3-hydroxy-C_14_-HSL. However, the corresponding *R. erythropolis* enzyme was not identified.

In general the enzyme class of oxidoreductases (EC 1) includes a large family called short-chain dehydrogenases/reductases (SDRs). For recent reviews see references [19]–[23]. Up to date, almost 47,000 examples are known [24]. The SDRs cluster into at least 300 distinct families [21]. SDRs have in general low sequence similarities but they all show a special folding pattern, the Rossmann fold motif for binding to their nucleotide cofactor, NAD(H) or NADP(H) [23]. The 3D-structures display highly similar α/β folding patterns with a central β-sheet. Based on distinct sequence motifs, functional assignments and classifications are possible. The active site is often composed of an Asn-Ser-Tyr-Lys tetrad and the catalytic mechanism usually is a hydrid and proton transfer from or to the nicotinamid and the active site tyrosine residue. The variable C-terminus provides substrate specificity. SDR enzymes play essential roles in a wide range of cellular activities including lipid, amino acid, carbohydrate, cofactor, hormone and xenobiotic metabolisms; but also in redox sensor mechanisms. Today 261 SDR structure entries are available at PDB and of these 159 have been classified as oxidoreductases (online search April 2011). Of these 55 are linked to bacteria but only 26 are unique and represent self-contained and genetically unmodified wildtype enzymes; and of these less than 10 appear to utilize NADP as a ligand. Furthermore no metagenome derived SDR structures have been deposited at PDB.

Within this work, we attempted to extend our knowledge on SDRs interfering with the bacterial quorum sensing mechanisms using a metagenome-based and structural approach. Therefore we identified a novel oxidoreductase, designated BpiB09, which was able to inactivate the 3-oxo-C_12_-HSL signaling molecule. The novel metagenome-derived enzyme strongly affected *P. aeruginosa* biofilm formation and other QS-related phenotypes. BpiB09 enzyme was crystallized and we solved the BpiB09 structure at a resolution of 2.4 Å together with the cofactor NADP. Structural inspection of BpiB09 reveals that it exhibits a typical SDR fold and the functional signatures of this family of proteins. Further transcriptome analysis of *P. aeruginosa* cells expressing *bpiB09* suggests that it has profound effects on QS related gene expression in this microbe.

## Results

### Screening a soil metagenome library for autoinducer I quenching proteins

**Figure 1:**
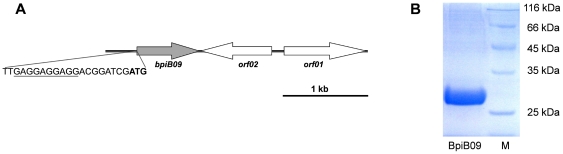
Key traits of the metagenome-derived clone Bio5. **A**) Physiological map of the insert of the metagenome derived clone Bio5 (3.1 kb, GenBank # EF530730.1); open reading frames identified are indicated as arrows in the direction of transcription. A potential Shine-Dalgarno sequence at the 5′-end of *bpiB09* is underlined; the potential translational start site of *bpiB09* is in bold; **B**) SDS-PAGE of recombinant BpiB09 after his-tag purification. Lane 1: 2 µg BpiB09; Lane 2: molecular weight marker (kDa).

Using a previously constructed metagenome library [25], a total of 8,000 metagenomic clones were tested three times for clones that were able to reduce the expression of the reporter gene in a screening system, which included the *A. tumefaciens* reporter strain NTL4 carrying a *traI*-*lacZ* reporter gene, X-Gal and 3-oxo-C_8_-HSL. The clone, designated Bio5, gave a consistent positive result in this screening assay. The insert of the detected clone Bio5 had a size of 3.1 kb. Sequencing identified three potential ORFs on Bio5 (Fig. 1A, GenBank entry EF530730.1). The ORF later proven to inhibit biofilm formation was labeled *bpiB09* for biofilm phenotype inhibiting gene and is highlighted in Fig. 1A. ORF1 was similar to chromosome segregation protein SMC in *Acidobacterium* sp. MP5ACTX8. The observed e-value for this similarity was 2e-126 (61% identity) and the similarity ranged over the entire protein; ORF2 was similar to hypothetical proteins found in *Hyphomonas neptunium* (3e-29, 42% identity); and BpiB09 was most similar to a possible short chain oxidoreductase (SDR) from *Acidobacterium capsulatum* (57% Identity) with an e-value of 8e-60.

In order to further characterize BpiB09, the metagenome-derived gene was amplified from the original metagenome clone DNA using PCR and specific primers. The resulting DNA fragment of approximately 720 bp was initially cloned into pBluescript SK+ then excised and cloned into the expression vectors pET-21a and pET-19b as described in experimental procedures. The correctness of the construct was verified by DNA sequencing. For recombinant expression the protein production was induced with 100 µM isopropyl-β-D-thiogalactopyranoside (IPTG) in LB medium in the *E. coli* strain BL21 DE3. The protein was purified from the soluble fraction using His-tag Ni TED purification. An SDS PAGE analysis indicated that the protein was homogenous with only minor contaminations through other proteins revealing a molecular mass of approximately 30 kDa (Fig. 1B), which corresponded well with the theoretical value of 27.4 kDa.

### BpiB09 affects QS-dependent phenotypes in *P. aeruginosa*

**Figure 2:**
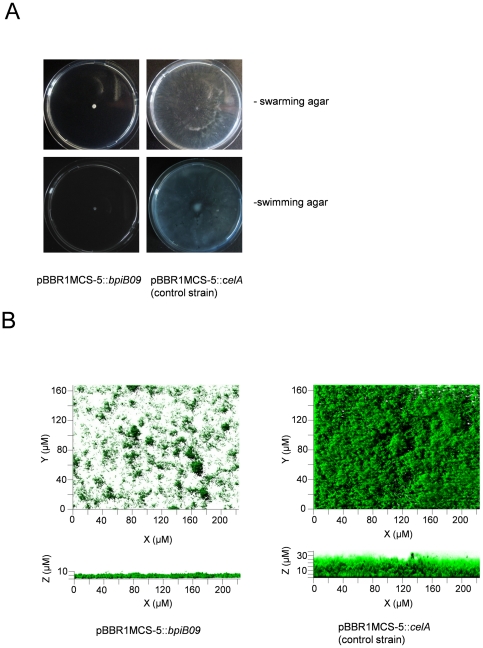
*BpiB09* expression causes reduced motility and poor biofilm formation in PAO1. **A**) Reduced swarming phenotypes (upper two panels) and reduced swimming phenotypes (lower panel) in *P. aeruginosa* carrying the pBBR1MCS-5::*bpiB09* or the control strain carrying the pBBR1MCS-5::*celA*; **B**) Decreased biofilm formation using the same constructs.

Because in *P. aeruginosa* motility is QS dependent [26], [27], the *bpiB09* gene was tested for its influence on motility in *Pseudomonas aeruginosa* PAO1. Therefore, the gene was cloned into the broad host range vector pBBR1MCS-5 and transformed into *P. aeruginosa* PAO1 (supplemental Table S1). As control we used a 2 kb cellulase gene, which was cloned into the same vector obtaining pBBR1MCS-5::*celA*. Previous studies had shown that this gene had no influence on PAO1 motility or biofilm formation ([25]; Fig. S1). Similar, the *Acidobacterium capsulatum* homologous gene was cloned into the same vector and used as a further control. The correctness of the inserts was verified by DNA sequencing. The *bpiB09* gene, when expressed in *P. aeruginosa*, significantly inhibited motility on swarming agar in comparison to the control (Fig. 2A, upper panels). Similar results were obtained when swimming motility was tested (Fig. 2A, lower panels). However, the *Acidobacterium capsulatum* homologous gene expressed on pBBR1MCS-5::*ACP_0942* in PAO1 did not affect motility (data not shown).

### BpiB09 affects *P. aeruginosa* biofilm formation

To further analyze the role of the metagenome-derived protein BpiB09 *in P. aeruginosa* biofilm tests were conducted using flow cells. For these tests we employed the PAO1 strain carrying the pBBR1MCS-5::*bpiB09* construct and the control strain carrying the pBBR1MCS-5::*celA* construct (supplemental Table S1). After 72 h the control strain, carrying the cellulase gene, had formed uniform biofilms with a thickness of 30–35 µm (Fig. 2B, right panel). At the same time, the tested *bpiB09* gene caused formation of rather thin biofilms with an average thickness of 5–10 µm (Fig. 2B, left panel). This indicates that the *bpi* gene inhibited biofilm formation in *P. aeruginosa*. Again the *Acidobacterium capsulatum* homologous gene did not affect PAO1 biofilm formation (data not shown).

### BpiB09 affects pyocyanin production in PAO1 and violacein production in *Chromobacterium violaceum*

**Figure 3 pone-0026278-g003:**
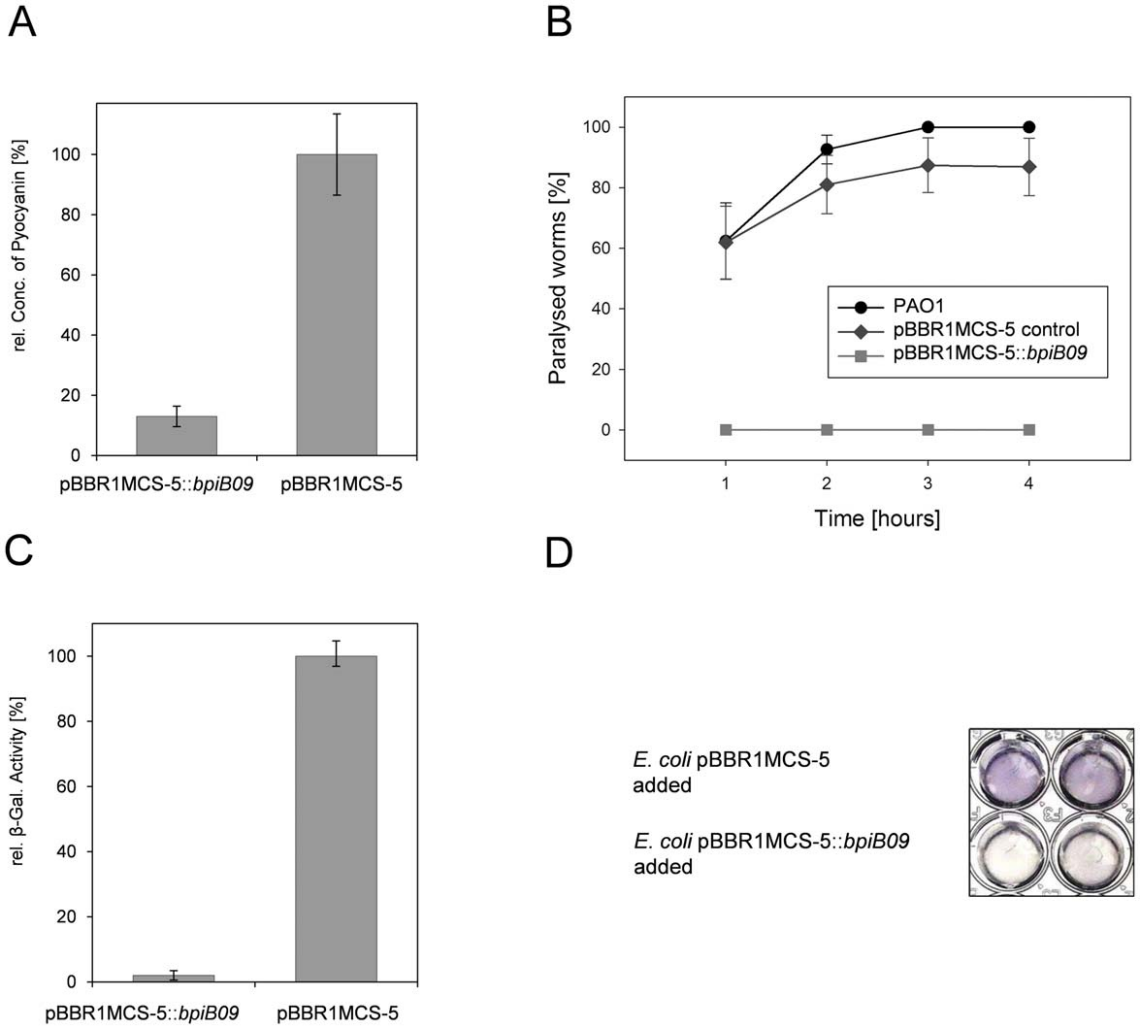
BpiB09 affects the QS-dependent pyocyanin production, *C. elegans* paralysis in PAO1 and decreases AHL response in *A. tumefaciens* AHL reporter strain. **A**) Decreased pyocyanin production. OD_520_ of pyocyanin extracts from culture supernatants of *P. aeruginosa* carrying pBBR1MCS-5::*bpiB09* and empty pBBR1MCS-5. Data represent mean values of at least five independent experiments. Bars indicate the standard deviations. **B**) *C. elegans* paralysis induced by PAO1 on BHI medium. Data represent mean values of eight independent assays per treatment (+/−) standard error. Incubation was carried out on BHI agar plates at room temperature. Per PAO1 strain eight replicate plates were assayed, each with 30 one-day-old adult *C. elegans*. GLM analysis revealed a significant PA strain effect between the PAO1 wildtype and pBBR1MCS-5::*bpiB09* (Likelihood ratio test, *χ^2^* = 132.04, df = 1, *P*<0.001), whereas there was no significant difference between the PAO1 wildtype and the empty vector control (Likelihood ratio test, *χ^2^* = 1.7 * 10^−7^, df = 1, *P*>0.99). PAO1, wild-type *P. aeruginosa* strain PAO1; PAO1 pBBR1MCS-5, control strain carrying an empty vector pBBR1MCS-5; PAO1 pBBR1MCS-5::*bpiB09*, expressing the *bpiB09* gene constitutively. **C**) Reduced ß-galactosidase activity (i.e. *traI-lacZ* activity) caused by spent medium of *P. aeruginosa* cultures after 16 hours of growth using the *A. tumefaciens* NTL4 reporter strain. Data represent mean values of at least five independent experiments. Bars indicate the standard deviations. **D**) Inhibition of quorum sensing dependent violacein production in *Chromobacterium violaceum* strain DSM # 30191. 200 µl *C. violaceum* culture incubated (16 h, 30°C) with 50 µl (10 µg/ml) of *E. coli* cell extract expressing an empty vector (pBBR1MCS-5) - upper row; or with cell extract of *E. coli* expressing the *bpiB09* gene in pBBR1MCS-5 - lower row.

Furthermore, it is known that pyocyanin production is QS dependent in *P. aeruginosa* [28]. Therefore, we tested the potential influence of the metagenome-derived *bpiB09* gene expression on pyocyanin production. Again the clone carrying the *bpiB09* gene in pBBR1MCS-5 showed no pyocyanin production in contrast to the controls where pyocyanin production was observed after 16 hours growth in LB medium at 37°C on the shaker (Fig. 3A). Additional tests using *C. violaceum* (DSM Nr. 30191) indicated that the recombinant and purified BpiB09 was highly active with respect to quorum quenching activities (i.e. the modification of the *C. violaceum* autoinducer I molecule) (Fig. 3D).

### Reduced paralysis of *Caenorhabditis elegans* by *P. aeruginosa* expressing *bpiB09*

The paralysis and killing of the nematode *Caenorhabditis elegans* by PAO1 is mediated by the QS dependent production of hydrogen cyanide [29]. We thus assayed the impact of the expression of *bpiB09* on the ability of *P. aeruginosa* PAO1 to paralyze *C. elegans*. While worms were rapidly paralyzed on PAO1 wildtype and PAO1 expressing the control plasmid, worms on the *bpiB09* expressing clone remained fully mobile and viable (Fig. 3 B).

### Reduced 3-oxo-C_12_-HSL autoinducer concentrations in supernatants of cells expressing *bpiB09* and activity of purified enzyme

Altogether, these tests suggested that the BpiB09 protein affected quorum sensing-dependent processes in *P. aeruginosa*. Thus we speculated that the protein directly interacts with the 3-oxo-C_12_-HSL molecule, the main form of autoinducer I in *P. aeruginosa*. To verify this hypothesis, supernatants of 18 h old cultures were tested using the *A. tumefaciens* strain NTL4, carrying a *traI*-*lacZ* fusion under the control of the AHL-inducible *traI* promoter as a reporter gene. These tests supported our hypothesis and clearly indicated a significantly reduced level of the 3-oxo-C_12_-HSL detected in *P. aeruginosa* cells expressing the *bpiB09* gene in comparison to the control strain (Fig. 3C).

**Figure 4:**
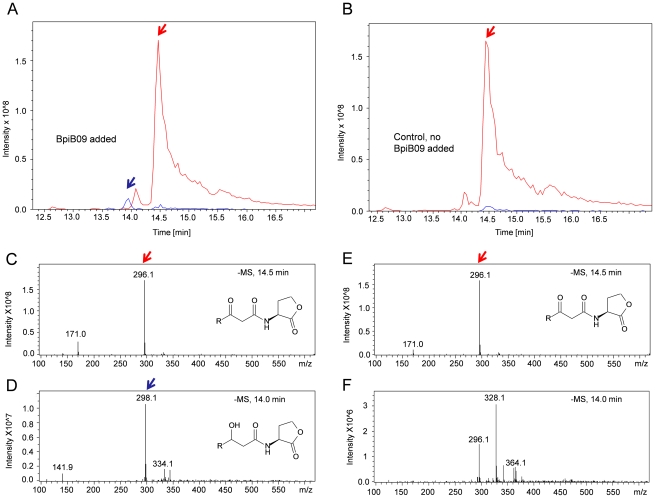
HPLC-MS profiles of 3-oxo-C_12_-HSL incubated with purified BpiB09. **A**) Analysis of 3-oxo-C_12_-HSL extract after incubation with BpiB09. Mass spectra show a [M-H]^−^ ion at an m/z of 296.1 for 3-oxo-C_12_-HSL (red arrows) and a [M-H]^−^ ion at an m/z of 298.1 for 3-hydroxy-C_12_-HSL (blue arrows). Extracted spectra recorded at t = 14.5 min (**C**) and at t = 14.0 min (**D**); **B**) Analysis of 3-oxo-C_12_-HSL extract after incubation with empty vector eluate (control). Extracted spectra recorded at t = 14.5 min (**E**) and at t = 14.0 min (**F**). For HPLC-MS-DAD-analyses see also supplemental material (Fig. S4).

Further HPLC-MS analyses of purified enzyme resulted in a turnover of 3-oxo-C_12_-HSL to 3-hydroxy-C_12_-HSL (Fig. 4). Mass spectra showed a [M-H]^−^ ion at an m/z of 296.1 for 3-oxo-C_12_-HSL (red arrow, Figs. 4A, 4B, 4C, 4E) and a [M-H]^−^ ion at an m/z of 298.1 for 3-hydroxy-C_12_-HSL (blue arrow, Figs. 4A, 4D). In the MS-MS spectrogram we also identified the masses of 112.0 and 141.9 corresponding to fragments of the 3-hydroxy-lacton (supplemental Fig. S4). The overall turnover of the substrate was rather low and probably less than 6% of the added substrate (5 mM 3-oxo-C_12_-HSL). This estimation is based on the peak intensities observed in MS analyses (Figs. 4C & 4D). These results suggested that 3-oxo-C_12_-HSL is probably not the native substrate of BpiB09.

### Genome-wide transcriptome analysis of *P. aeruginosa* cells expressing *bpiB09*

To further elucidate the impact of *bpiB09* expression on PAO1, we initiated a genome-wide transcriptome analysis using 454-cDNA sequencing. Within this study we compared the cDNA levels of cells that were grown for 5 hours in LB medium and that carried the *bpiB09* gene on pBBR1MCS-5::*bpiB09* under the control of its native promoter. As a control we used the same strain but carrying an empty vector. A careful evaluation of the data suggested that the *bpiB09* gene in PAO1 was expressed at a significant level, comparable to the expression of the ribosomal protein genes *rplR*, *rpsL* and *rplM*, belonging to the 70 most abundant expressed genes. Almost equal hits were observed for these genes and *bpiB09* (data not shown). Thus we assume that probably sufficient BpiB09 protein was formed within the cell. Overall the level of gene expression of housekeeping genes (for example *recA*, *rpoD*, *gyrA*, *gyrB* and *dnaA*) was similar and differed not more than 2.5-fold in the reference samples and the PAO1 carrying the *bpiB09* gene.

Our data also showed that as a result of the *bpiB09* expression a significant fraction of PAO1 genes were differentially regulated in both strains. Within our analysis we mainly focused on those genes that are possibly linked to QS according to references [30]–[32]. Those genes that were at least 10-fold down-regulated were included in Table 1 (gene/ORF names taken from *Pseudomonas* genome project [33]). Among the genes significantly down-regulated we identified the *lasI*, *lasB*, *rhlI*, *hcnA* and *pqsA/B/C/D* genes, thus including autoinducer synthesis genes for both AHLs as well as for the Pseudomonas quinolone signal (Table 1). In addition a significant number of genes and ORFs were detected that had been linked to QS-phenotypes in PAO1 and that were less than 10-fold but at least 4-fold altered in their expression level. Altogether these were 80 genes/ORFs and among those we found the *hcnB* and *hcnC* genes involved in hydrogen cyanide synthesis, the *aprD* and *aprE* genes involved in alkaline protease secretion as well as *lecB* and *lasA*. Although the majority of the flagellar genes was not affected by *bpiB09* expression, the *fliC* gene, encoding the major flagellin, was strongly (>20-fold) repressed. Also the transcription of *flgD*, *flgE* and *flgF* was at least tenfold down regulated (Table S2).

**Table 1:** QS-controlled and ≥10-fold repressed genes/ORFs in PAO1 expressing *bpiB09* a.

ORF^b^	Gene	Description	Change in expression (fold)^c^
PA0007		hypothetical protein	−12
PA0122		hypothetical protein	−12
PA0179		two-component response regulator	ND^d^
PA0572		hypothetical protein	−12
PA0996	*pqsA*	coenzyme A ligase	−55
PA0997	*pqsB*	hypothetical protein PqsB	−130
PA0998	*pqsC*	hypothetical protein PqsC	−46
PA0999	*pqsD*	3-oxoacyl-(acyl carrier protein) synthase III	−94
PA1003	*mvfR*	transcriptional regulator	−12
PA1130	*rhlC*	rhamnosyltransferase 2	−12
PA1248	*aprF*	Alkaline protease secretion AprF precursor	−37
PA1250	*aprI*	alkaline proteinase inhibitor	−13
PA1432	*lasI*	autoinducer synthesis protein	−150
PA1784		hypothetical protein	−30
PA2069		carbamoyl transferase	−49
PA2193	*hcnA*	hydrogen cyanide synthase	−24
PA2300	*chiC*	chitinase	−15
PA2303	*ambD*	hypothetical protein	−79
PA2304	*ambC*	hypothetical protein	−30
PA2591	*vqsR*	transcriptional regulator	−16
PA2592		periplasmic spermidine -binding protein	−20
PA3328		FAD-dependent monooxygenase	ND
PA3329		hypothetical protein	−24
PA3331		cytochrome P450	−22
PA3332		hypothetical protein	−29
PA3333	*fabH2*	3-oxoacyl-(acyl carrier protein) synthase III	−35
PA3334		acyl carrier protein	−34
PA3335		hypothetical protein	ND
PA3476	*rhlI*	autoinducer synthesis protein	−16
PA3478	*rhlB*	rhamnosyltransferase chain B	−15
PA3479	*rhlA*	rhamnosyltransferase chain A	−25
PA3724	*lasB*	elastase	−840
PA3906		hypothetical protein	−50
PA3907		hypothetical protein	−15
PA3908		hypothetical protein	−20
PA4209	*phzM*	phenazine-specific methyltransferase	−15
PA4211	*phzB1*	phenazine biosynthesis protein	−16
PA5059		transcriptional regulator	−13

a Genes/ORFs listed as being QS-regulated refer to the studies by Schuster *et al.* [31], Wagner *et al.* [32] and Hentzer *et al.* [30]. Listed are genes common to all three studies, which were repressed in our transcriptomic analysis with a magnitude of change of ≥10fold. Additionally *lasI* and *rhlI* are enlisted, which could not be measured in the three mentioned transcriptomic analyses, because of the use of a *lasI-rhlI*-mutant strain.

b Gene/ORF numbers, names and descriptions are derived from the *Pseudomonas* genome project [33].

c Changes in gene expression (rounded to two significant figures) of *P. aeruginosa* PAO1 expressing *bpiB09* compared to a control-strain with empty vector. The displayed values are the averages out of two completely independent transcriptomic analyses, samplepoint 5 h.

d ND, no transcripts detectable.

Altogether, these findings supported the observations made with respect to the observed phenotypes of cells expressing the *bpiB09* gene (i.e. reduced motility, biofilm formation, pyocyanin production, autoinducer production and *C. elegans* pathogenicity; Figs. 2 and 3). Genes that were at least 10-fold induced were included in Table S3.

Further our data also suggested that the overall expression of the synthesis genes of the different autoinducers was abolished or strongly down-regulated. Thus expression of *bpiB09* in PAO1 clearly affects transcription of known QS-regulated genes and ORFs and has therefore strong impact on the physiology of this microbe.

### Structural analysis

Sequence based classifications assign BpiB09 to the large superfamily of short-chain reductases (SDR). The SDR superfamily consists of over 47,000 variants, with residue identities of 20–30% [34]. This superfamily of enzymes can be subclassified at a first level into 5 families based on chain lengths and conserved sequence motives. Based on those classifications, BpiB09 belongs to the so-called classical SDR family. The presence of an arginine in the Gly-motif (R16) and at the first position after the second beta strand (R38) assigns BpiB09 into subfamily cP3 [35].

BpiB09 was crystallized and a complete data set was collected to 2.4 Å resolution at beamline X13 at EMBL Hamburg/DESY. Data collection and refinement statistics are summarized in Table 2. The structure was solved by molecular replacement using PDB structure 2EHD as a template. Crystals belong to space group I422 with unit cell dimensions of *a* = *b* = 243.2 Å, and *c* = 151.5 Å.

**Table 2:** Data collection and refinement statistics.

Data collection	BpiB09
Beamline	X13 EMBL/DESY
Space group	I422
Wavelength (Å)	0.8123
Detector distance (mm)	229.63
Cell dimensions	
*a*, *b*, *c* (Å)	243.16 243.16 151.54
Resolution (Å)	47.7-2.42 (2.55-2.4)
Number of reflections	481,415(61,551)
*R*_merge_	5.3(70.0)
*R_pim_*	2.4(33.2)
*I/σI*	24.4(2.3)
Completeness (%)	98.6(95.9)
Multiplicity	5.6(5.1)
**Refinement**	
No. reflections	85,193
*R*_work_/*R*_free_	19.3/22.2
No. atoms	
Protein	6,418
Ligand/ion	163
Water	252
B-factors	
Protein	59
Ligand/ion	101.2
Water	55
R.m.s deviations	
Bond lengths (Å)	0.015
Bond angles (°)	1.457

**Figure 5:**
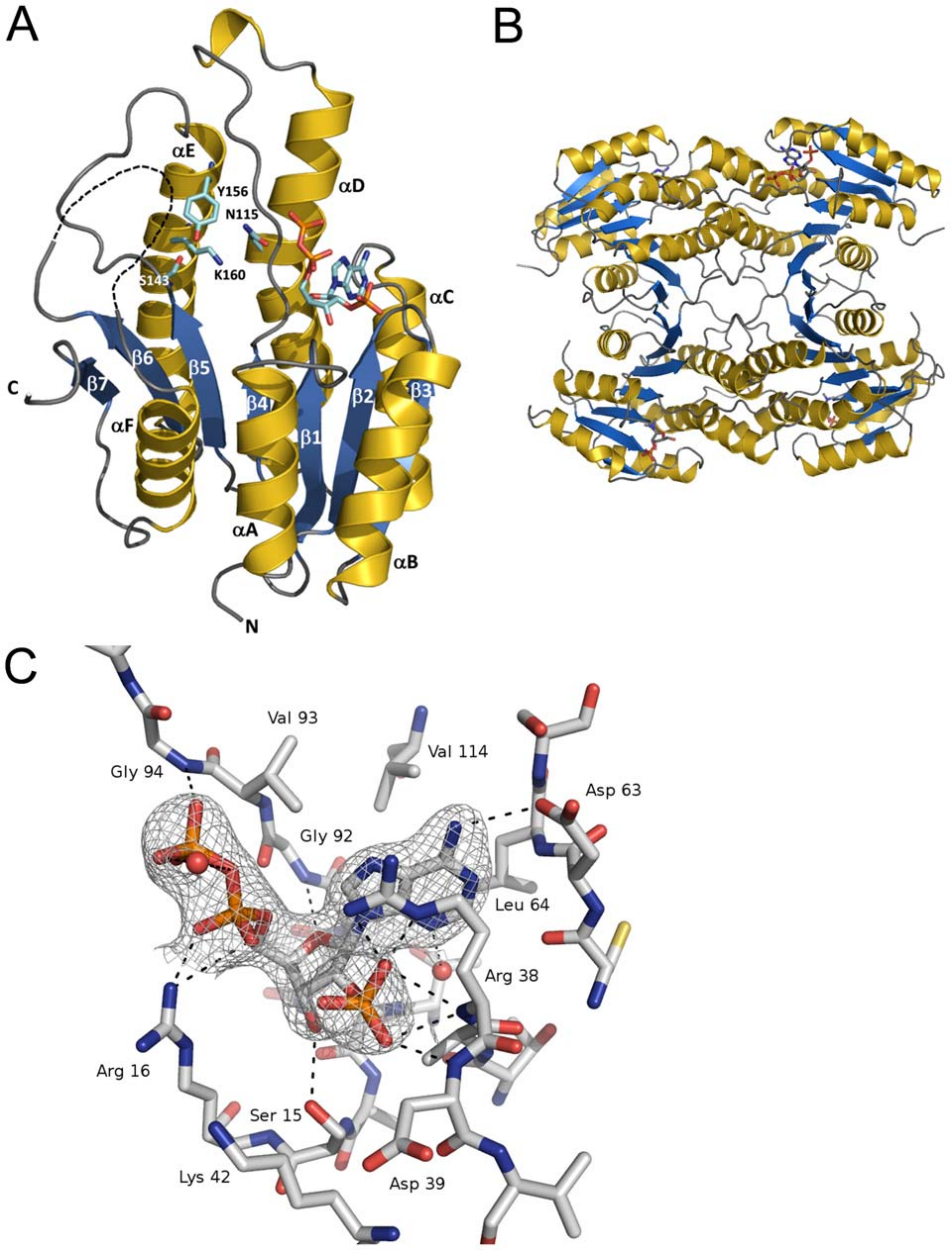
The crystal structure of BpiB09 in complex with NADP^+^. **A**) BpiB09 monomer, β1–7 indicate the β sheets and α 1–6 indicates the alpha helices. Catalytic residues are indicated together with the NADP-binding site residues. **B**) Tetrameric arrangement of BpiB09 as probably present in solution together with the bound cofactor NADP. **C**) Cofactor binding in BpiB09. Supposed hydrogen bonds are shown as orange broken lines. The electron density at the NADP molecule is contoured at 1 σ.

**Figure 6:**
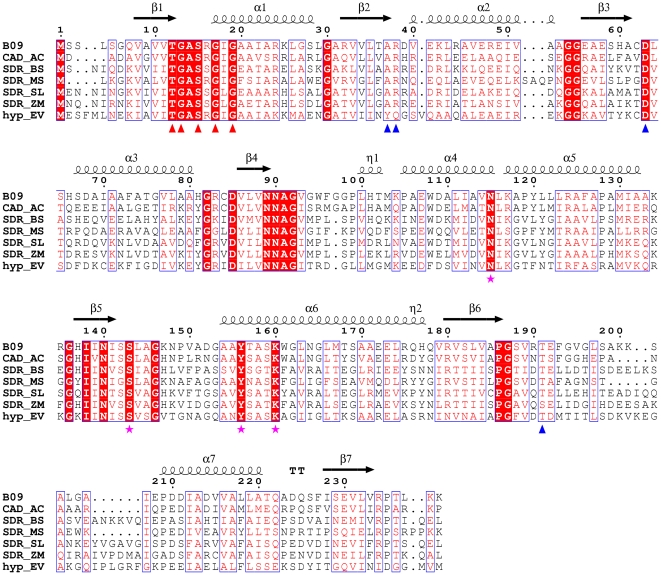
Structure-related functional sequence conservation between BpiB09 and homologous proteins. Shown above the alignments are elements of the secondary structure of BpiB09. The numbering shown is from BpiB09. *Red triangles* indicate the dinucleotide-binding motif. *Blue triangles* indicate critical cofactor binding residues. *Purple stars* represent putative catalytic residues located at the corresponding positions of the conserved catalytic residues N115, S143, Y156, and K160, respectively, in the SDR family proteins. Strictly conserved residues are highlighted with *red boxes*. Biological sources and GenBank accession codes for the sequences are as follows: CAD_AC, *Acidobacterium capsulatum* (YP_002754051.1); SDR_BS, *Bacillus subtilis* subsp. Spizizenii (ZP_06875312.1); SDR_MS, *Meiothermus silvanus* (YP_003684578.1); SDR_SL, *Spirosoma linguale* (YP_003387009.1); SDR_ZM, *Zymomonas mobilis* subsp. Mobilis ZM4 (YP_163311.1); hyp_EV, *Eubacterium ventriosum* (ZP_02027436.1). Sequence alignments were assembled using T-COFFEE software (http://www.ebi.ac.uk/Tools/t-coffee) and visualized using ESPript software (http://espript.ibcp.fr/ESPript/cgi-bin/ESPript.cgi).

BpiB09 assumes a typical Rossmann fold with a central beta sheet flanked by helices αA αB and αF on one side and αC, αD and αE on the other side (Fig. 5A). The strand topology is 3-2-1-4-5-6-7 with a crossover connection linking strands 3 and 4. This creates a characteristic nucleotide binding site across the topological switch point between strands 1 and 4. A Gly-motif (TGxxxGxG, Fig. 6) at the N-terminal region delineates the binding site of the adenosine half of the dinucleotide co-factor (Fig. 5C). The reactive nicotinamide half is expected to bind in the variable C-terminal region close to the conserved active site tetrade Asn^115^-Ser^143^-Tyr^156^-Lys^160^ [36].

The active substrate binding site of BpiB09 can unambiguously be located in the variable C-terminal region by the presence of a catalytic tetrad. Immediately adjacent to the active site is a 21 residue long loop connecting strand β6 and helix αF. Within this loop 16 residues are disordered (res. 191 through 206) (Fig. 5A). This leaves the active site wide open and suggests that the loop closes like a lid once substrate binding has occurred as observed in other structures of SDRs. Closure would shield the active site from bulk solvent during catalysis and prevent unproductive hydride transfer. The active site architecture suggests a reaction mechanism for BpiB09 analogous, but reverse to 3β/17β-hydroxysteroid dehydrogenase [36]. In BpiB09 the transfer of the 4-pro-S hydride from nicotinamide onto the substrate would be accompanied by a proton relay through the side chains of Tyr156, the nicotinamide ribose hydroxyl, Lys160 and a water molecule, which is stabilized by the main-chain carbonyl of Asn115 [22].

Most SDRs are either homodimeric or homotetrameric. Crystals of BpiB09 contain four copies per asymmetric unit, which correspond to two halves of a homotetramer based on the arrangement in other homotetrameric SDRs (Fig. 5B). Tetramerization of BpiB09 had already been observed during purification by gel permeation chromatography (data not shown) and was further confirmed by analytical ultracentrifugation (supplemental Fig. S2A). The dimerization interface within the asymmetric unit involves an extended beta-sheet, which aligns strand β7 of two protomers in an anti-parallel fashion (A and D monomer). This interface covers an area of 1260 Å^2^. The second interface, which relates the dimer of dimers across a crystallographic dyad comprises a four-helix bundle formed by helices α4 and α5 and their corresponding symmetry related helices (dimer AD and A′D′). This interface consists of 1550 Å^2^. This oligomeric arrangement corresponds to a D_2_ symmetry. Interestingly, removal of the C-terminal strand β7, which is not part of the classical Rossmann fold, abrogates not only tetramerization but results in the formation of monomers in solution (supplemental Fig. S2B). Likewise, when mutating residues G162Y and D109K in the helical interface again the monomeric form was observed (supplemental Fig. S2C). This indicates that tetramer formation is cooperative. None of the individual interfaces are of sufficient strengths to support even the dimeric form.

### Cofactor binding to BpiB09

SDRs can utilize NAD or NADP as co-enzymes. Both, the presence of characteristic sequence motives and of a partial nicotinamide adenine dinucleotide phosphate unambiguously denote BpiB09 as an NADP-dependent oxidoreductase. During refinement of the structure residual electron density appeared at the topological switch point of BpiB09 which was modeled as a partial NADP^+^. The absence of continuous electron density after the beta-phosphate of the nucleotide indicates that the nicotinamide was not tightly bound by the substrate free form of BpiB09. These Crystals obtained from the original sample had not been exposed to NADP^+^ after purification from *E. coli*. This indicates that the heterologously expressed enzyme scavenged the coenzyme from its expression host. Subsequent attempts to improve the electron density of the co-enzyme by adding NADP^+^ prior to crystallization resulted in a higher occupancy of the bound NADP^+^ but did not change the loose binding mode (Fig. 5C). As indicated earlier, the active site of BpiB09 is exposed to the solvent and the 21 residue long loop likely covers the site only after the substrate has bound, as observed in pertinent SDRs. The loops positional flexibility likely prevents NADP^+^ from binding in a reaction compatible manner. There is residual electron density close to Lys^160^ and Lys^147^, which defies interpretation due to its poor quality. Conceivably, the nicotinamide and ribose bind in several conformations and contributes patchy electron density.

In each of the dimers, related by the extended beta-sheet, one monomer shows better density for NADP^+^ indicating tighter binding and higher occupancy.

## Discussion

We have identified a novel NADP-dependent oxidoreductase from a soil metagenome. To the best of our knowledge this is the first example of such an enzyme derived from the metagenome that has been characterized in detail. The protein was designated BpiB09 because it caused a **b**iofilm **p**henotype **i**nhibition in the opportunistic pathogenic microorganism *P. aeruginosa*. Before this study, little was known on the details of AHL-converting oxidoreductases at the molecular level that are involved in biofilm inhibition and other QS-dependent phenotypes. At least two AHL quorum-sensing signals (i.e. 3-oxo-C_12_-HSL & C_4_-HSL) are involved in the regulation of surface translocation and biofilm differentiation in *P. aeruginosa* [26], [37]. Furthermore the 3-oxo-C_12_-HSL and C_4_-HSL are of importance for the production of virulence factors, such as proteases, phospholipases and cyanide [38]. It is well known, that mutants defective in AHL signaling can be attenuated in virulence when assayed on *C. elegans* and a mouse model of pneumonia [39]–[41]. To test the effects of the metagenome derived-AHL-oxidoreductase expression on quorum-controlled phenotypes, the *bpiB09* gene was brought into *P. aeruginosa* PAO1. Within this work the expression of the *bpiB09* gene in *P. aeruginosa* significantly reduced the accumulation of 3-oxo-C_12_-HSL in the growth medium (Fig. 3C). This, in turn, resulted in the decreased production of quorum-controlled virulence factors, reduced swarming and swimming motility and attenuated virulence on *C. elegans* (Figs. 2AB & 3AB). These data are consistent with earlier reports on effects of the expression of quorum quenching genes in PAO1 [7], [13], [25]. It may be interesting to notice that both swarming and biofilm formation are strongly reduced, whereas other work has demonstrated an inverse regulation of these phenotypes [42].

A genome-wide transcriptome study suggested that the *lasI* and *rhlI*-genes were not or only weakly transcribed in *P. aeruginosa* cells carrying copies of *bpiB09* in comparison to a control strain (Table 1). The *P. aeruginosa* genome encodes for about 5,570 open reading frames [43] and to date a few studies have been published that analyzed different expression profiles on a whole genome scale and with respect to QS-regulated processes in *P. aeruginosa* strain PAO1 [30]–[32]. Altogether, these studies suggest a set of at least 85 genes to be involved in QS-depended processes in PAO1. Within this framework, we have analyzed the transcriptome of PAO1 planktonic cells expressing the *bpiB09* gene compared to cells harbouring an empty control vector. Our transcriptome data revealed that the expression of the *bpiB09* gene caused significantly reduced (≥10-fold) gene expression of at least 38 QS-dependent genes in PAO1. The identified genes matched mostly the genes and ORFs identified by the above mentioned studies. However, our data also suggested that the expression of the *bpiB09* gene had a strong impact on all QS-related processes in PAO1 and that mainly the synthesis of the different autoinducers was abolished.

These data were in line with our chemical analyses and the observed physiological phenotypic data (Figs. 2–4). It is noteworthy that no *C. elegans* paralysis was detected in PAO1 expressing *bpiB09* (Fig. 3B), while the expression of an acylase in PAO1 could significantly reduce but not eliminate paralysis [13]. The expression of *bpiB09* had no significant impact on growth and biomass production of *P. aeruginosa*. Optical densities did not differ more than OD 0.1 after 5 hours of growth and OD 0.3 after 20 hours of growth when the cells were grown on liquid LB medium, growth was only slightly enhanced by less than 10%.

NCBI Conserved Domain Search (http://www.ncbi.nlm.nih.gov/Structure/cdd/wrpsb.cgi) displayed sequence similarity of BpiB09 to the NADB_Rossmann super family (Rossmann-fold NAD(P)(+)-binding proteins) and to 3-ketoacyl-(acyl-carrier-protein) reductases. This is also confirmed by the structure of BpiB09 and its similarity to other SDR family structures based on a DALI search of the PDB. All of the similar proteins in accordance with Prosite (http://www.expasy.ch/prosite/) contain the short-chain dehydrogenases/reductases family signature (ADH_SHORT): [LIVSPADNK] - x(9) - {P} - x(2) - Y - [PSTAGNCV] - [STAGNQCIVM] - [STAGC] - K - {PC} - [SAGFYR] - [LIVMSTAGD] - x - {K} - [LIVMFYW] - {D} - x - {YR} - [LIVMFYWGAPTHQ] - [GSACQRHM]. Cofactor binding requires the formation of specific hydrogen bonds and van der Waals contacts in a pocket between the first strand and the subsequent helix of the Rossmann-fold topology. Typically, this pocket contains a consensus binding motif in classical SDRs similar to TGXXXGXG, in which the first 2 glycines participate in the NAD(P)-binding. Furthermore, proteins in this family contain a C-terminal extension, which is responsible for specifically binding a substrate and catalyzing a particular enzymatic reaction. Substrate specificity of members of the family of SDRs basically is governed by the rather non-conserved C-terminal 50–60 amino acids. Despite a detailed analysis of the C-terminal region of BpiB09 and the observation that it reduces 3-oxo-C_12_-HSL to some extent, we have no clear understanding on the natural substrate of this enzyme.

**Figure 7:**
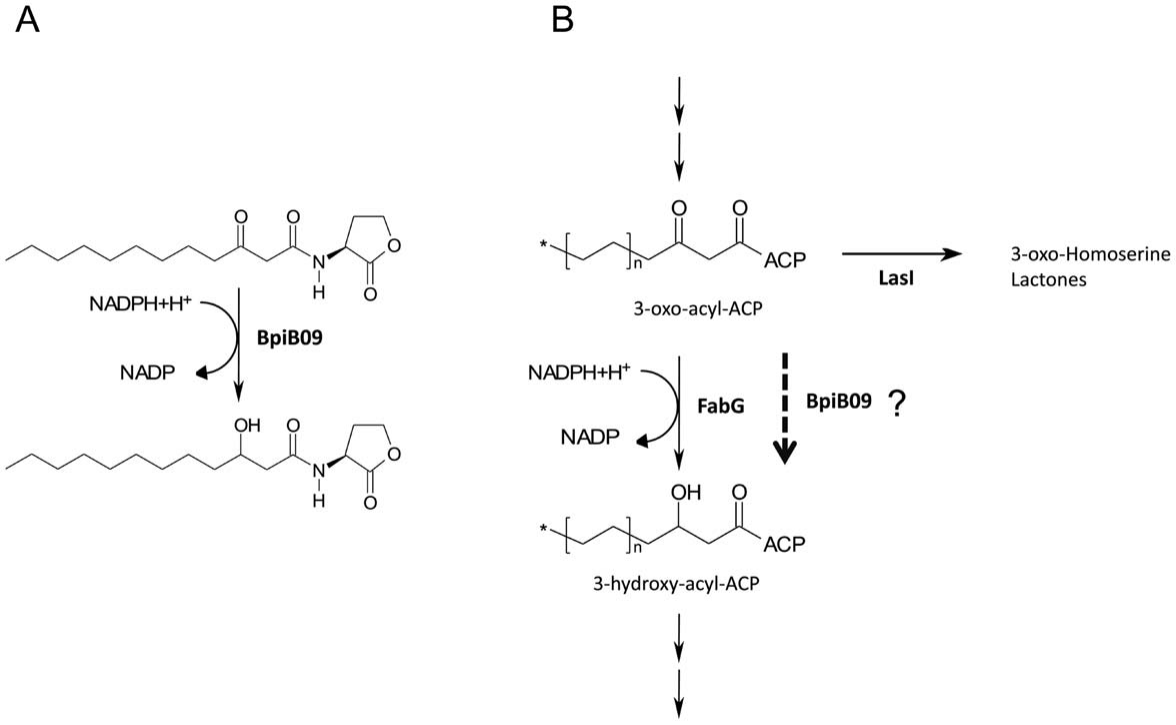
Verified and proposed BpiB09 activity in *P. aeruginosa* cells. **A**) Activity verified by HPLC-MS. The 3-oxo-group of the autoinducer is reduced to a 3-hydroxy-group. **B**) Proposed additional activity in the synthesis pathway of homoserine lactones. Due to the similarity of the verified substrate 3-oxo-C_12_-HSL to intermediates of the fatty acid cycle we propose the depicted additional activity.

However, our structural and transcriptome data suggest that BpiB09 perhaps has two functions in the cell: First it reduces the 3-oxo-C_12_-HSL molecules (Figs. 4 & 7A) and thereby strongly reduces the signaling molecule; and secondly it interferes with the synthesis of the autoinducers itself by reducing the free 3-oxo-acyl-ACP in the cell (Fig. 7B). This would in turn result in a decreased substrate availability for the PAO1 autoinducer synthase (LasI) and consequently result in reduced levels of formed autoinducers and in an absence of autoinduction of *lasI* transcription. Unfortunately, we have no chemical data available to prove this hypothesis. However, the almost complete lack of *lasI* and *rhlI* expression support this hypothesis. Furthermore BpiB09 acts as SDR on 3-oxo-C_12_-HSL (Figs. 4 & 7A) and due to the structural similarity of the substrate it is very well possible that the protein also acts on the 3-oxo-acyl-ACP available in the cell (Fig. 7B).

Within this framework, it is worthwhile noting that BpiB09 is a novel SDR and no enzyme with a similar activity has been described yet. A phylogenetic analysis suggested that the protein is different from known SDRs. The most similar proteins found in GenBank were SDRs from *Acidobacterium* and from *Koribacter* (supplemental Fig. S3). However, the overall similarity was not higher than 58% suggesting that BpiB09 originates from a not yet cultivated microbe. Since the protein was derived from a metagenome it will not be possible to speculate on the original host. It appears that BpiB09 represents probably the first NADP-dependent SDR derived from a non-cultivated microbe whose structure is deposited at PDB.

Concerning a potential application of the protein for the prevention of microbial biofilms, we can currently only speculate about the success of such an attempt. However, taking into account the strong phenotypes observed in our biofilm tests (Fig. 2B) it might indeed be possible to use the protein for quenching the QS signal and thereby suppressing bacterial biofilm formation at a very early stage. In fact, several examples have been published demonstrating that the expression of quorum quenching enzymes can result in the reduction of pathogenicity and virulence [44]–[46]. Since BpiB09 requires NADPH as a cofactor it might however, require the additional supply of the cofactor at sufficiently high concentrations for the reduction of the respective autoinducer molecules. Current work needs to assess the feasibility of this approach by immobilization of BpiB09 on a catheter or other surfaces. Those immobilized proteins can then be used to analyze the role of the BpiB09 protein on developing *P. aeruginosa* or mixed species microbial biofilms.

## Materials and Methods

### Bacterial strains, plasmids and culture conditions

Bacterial strains and constructs used in this work are listed in supplemental Table S1. *E. coli* and *P. aeruginosa* PAO1 were maintained in LB medium [47] at 37°C. For clones containing the phagemid vector pBK-CMV kanamycin (final concentration of 25 µg/ml) was added, for clones containing the vector pBluescriptSK+ ampicillin (100 µg/ml) and for clones containing the broad host range vector pBBR1MCS-5 gentamycin (10 µg/ml in *E. coli* or 50 µg/ml in *P. aeruginosa*) was added. Plasmid transformation in *E. coli* was done following standard electroporation protocols, heat shock or conjugation protocols [47]. *P. aeruginosa* was transformed using electroporation [48]. *A. tumefaciens* NTL4 [49], carrying a *traI-lacZ* promoter fusion on vector pCF372 [50] and extra copies of *traR* on vector pCF218 [51] and *A. tumefaciens* KYC6 [51], which is a natural overproducer of homoserine lactones, were grown in LB or AT medium [52] containing 0.5% glucose per liter at 30°C. For *A. tumefaciens* NTL4 spectinomycin (final concentration 50 µg/ml) and tetracycline (final concentration 4.5 µg/ml) were added. Biofilm experiments in flow chambers were performed as previously published [7], [25].

### Screening for quorum sensing inhibiting clones

The Screening was performed as previously published [7], [25]. The positive clones were verified at least three times. In addition, we used *C. violaceum* to also verify the observed results. For these tests a cell free extract was prepared of the Bio5 clone and of *E. coli* XL1 blue containing an empty vector. The protein concentration was determined and adjusted to 10 µg/µl. An overnight culture of *C. violaceum* was prepared and 50 µl were mixed with 50 µl of the cell extracts and incubated overnight at 30°C. Absence or impairment of purple coloration indicated quorum quenching. The insert of the single positive clone Bio5 was sequenced using automated sequencing technologies (MegaBACE 1000 System, Amersham Bioscience). Gaps were closed by primer walking. All potential ORFs were analyzed using blastX or blastP (NCBI). Sequences were deposited at GenBank with the accession number EF530730.1. For the detection of the respective ORFs involved in quorum sensing inhibition subcloning was employed. The potential quorum sensing inhibiting ORF was amplified using the primer combination given in supplemental Table S4. Clones were assayed using the *A. tumefaciens* reporter strain described above and using the *P. aeruginosa* motility assays [7], [25].

### Motility testing

The motility of *P. aeruginosa* was examined using swimming and swarming agars in petri dishes. *Pseudomonas* swarming agar contained M9 medium [47] without NH_4_Cl, and included 0.05% glutamic acid. This was solidified with 0.5% Eiken agar (Eiken, Tokyo, Japan). Swimming plates contained M9 medium with NH_4_Cl but no glutamic acid, and were solidified with 0.3% agar. For motility tests 1×10^7^ cells in 1 µl of an overnight *P. aeruginosa* PAO1 culture were applied to the middle of the agar plate. The plates were incubated at 37°C for 24 h. Swarming and swimming were documented by photography.

### β-galactosidase reporter tests using *A. tumefaciens* NTL4 (pCF218/pCF372)

To assay the AHL concentration in *P. aeruginosa* culture supernatants, a 20 h old *P. aeruginosa* culture (5 ml) was pelleted. The supernatant was sterile filtered and 8 µl were added to 5 ml of a freshly inoculated (1%) *A. tumefaciens* NTL4 (pCF218/pCF372) culture in AT medium. This strain harbors multiple copies of the AHL receptor gene *traR* detecting AHLs as well as a *lacZ* gene containing a promoter that gets activated by TraR-HSL complexes. The culture was grown at 28°C to an OD of approximately 0.6, the OD_600_ was measured, and 1 ml of the cells was lysed with toluol. The cell extract containing active β-galactosidase was incubated with o-nitrophenyl-β-D-galactopyranoside (ONPG) as a substrate. The cleavage product o-nitrophenol was then detected at 420 nm using a photometer. The measured values for OD_420_ were divided by the values for OD_600_. Tests were usually repeated at least 3–5 times and mean values were calculated. Control samples lacking AHLs were assayed in parallel and those values were subtracted from the data obtained with the supernatants containing AHLs.

### Cultivation of *P. aeruginosa* PAO1 biofilms in flow chambers

Biofilms were cultivated in two-channel flow cells constructed of V10A stainless steel. The individual channel dimensions were 3 mm×8 mm×54 mm. The substratum consisted of standard borosilicate glass coverslips (24 mm×60 mm, thickness 0.17 mm) that were fixed on the upper and lower side of the stainless steel flow chamber (1.3 ml total volume), using additive-free silicone glue. The assembled flow cells with Tygon tubing (inner diameter 2.79 mm) attached to the outlets of each channel were sterilized by autoclaving at 121°C for 15 min. All experiments were performed at 30°C. Prior to inoculation, the flow chamber was rinsed with mAPM medium for 5 h at a flow rate of 20 ml/h, using a multi-channel Ismatec IPC-N peristaltic pump. For inoculation, bacteria from a 16 h culture in LB medium was washed once and then resuspended in mAPM medium; each channel of the flow cell was inoculated with 5 ml of the cell suspension adjusted to approximately 10^7^ cells/ml. After arresting the medium flow for 1 h, it was resumed at a rate of 20 ml/h (83.3 cm/h), corresponding to a flow with a Reynolds number of 0.73. The residual time of the bacteria in the flow chambers was approximately 4.5 min. After 72 h, biofilm cells were stained with SYTO 9 (Invitrogen, Darmstadt, Germany) by injecting 2 ml of SYTO 9 solution (0.2 µl SYTO 9/ml mAPM), and viewed by fluorescence microscopy.

### Fluorescence imaging analysis of *P. aeruginosa* PAO1 biofilms in flow chambers

Visualization of flow-cell biofilms was performed using a Zeiss Axio Imager 2 fluorescence microscope (Zeiss, Jena, Germany). Images were obtained with a Zeiss LD Achroplan 40×/0.60NA objective. Three-dimensional image stacks of 72 h old flow-cell biofilms stained with SYTO 9 were recorded at an excitation wavelength of 470 nm in combination with an emission filter BP 525 nm. Digital image acquisition, analysis of the optical thin sections and three-dimensional reconstructions were performed using the Zeiss AxioVision software (version 4.8.1). Tests were verified in at least three independent experiments.

### Protein purification

For this purpose, *bpiB09* was cloned into the expression vector pET-19b and transferred to *E. coli* BL21 DE3. This strain was grown at 30°C to OD_600_ 0.6–1.0 in LB cultures containing ampicillin (100 µg/ml). Expression was induced by addition of IPTG to a concentration of 100 µM and carried out for 16 h at 17°C. Cells were harvested and resuspended in 1× LEW buffer (Macherey-Nagel, Düren, Germany) prior to cell disruption through sonication (Sonicator UP 200S, Hielscher, Germany) at 50% amplitude and cycle 0.5 for 5 min or using a French pressure cell. After centrifuging at 13,000 rpm and 4°C for 30 min the crude cell extract could be used for testing or was then purified using Protino columns from Macherey-Nagel (Düren, Germany) following the manufacturer protocol. The levels of protein purity, as well as the molecular mass, were determined by SDS-gel electrophoresis.

### HPLC-MS analysis

For chemical analysis 2 µmol 3-oxo-C_12_-HSL (Sigma-Aldrich, Heidelberg, Germany) (4 mM final concentration in 0.5 ml total volume) was mixed with 1 mM NADPH and 100 µg purified BpiB09 protein in 100 mM potassium phosphate buffer pH 7.0 with 20% DMSO as cosolvent and incubated for 16 h at 30°C. After incubation, the resulting mixtures were extracted twice with ethyl acetate (total of 2 volumes) and the combined organic layers were concentrated in vacuo. For HPLC analysis each extract was dissolved in methanol (5 mg/ml). HPLC-MS-DAD (coupled high performance liquid chromatography mass spectrometry-diode array detector) analysis of the solutions was performed using a Nucleosil 100 C18, 3 µm (100×2 mm) column, an Agilent 1200 LC-System and LC/MSD Ultra Trap System XCT 6330 with the software 6300 Series Trap Control Version 6.1. A gradient program with solvent A (water with 0.1% formic acid) and B (acetonitrile with 0.06% formic acid) was used to detect the 3-oxo-C_12_-HSL and the reduced product (retention times 14.5 min and 14.0 min, respectively): gradient from 0% B to 100% B in solvent A in 20 min, 3 min 100% B, post-time 5 min 0% B (total: 28 min program) at a flow rate of 400 µl/min. HPLC-MS and HPLC-MS/MS mass analysis were recorded using negative ionization. As reference the commercially available 3-hydroxy-C_12_-HSL [Sigma-Aldrich, Heidelberg, Germany] was measured.

### Measurement of the pyocyanin production in PAO1 cultures

Analysis of the pyocyanin production was performed as previously published [53]. Cultures were grown for 16 h with aeration at 37°C prior to the pyocyanin measurements.

### cDNA library construction and transcriptome analysis of *P. aeruginosa* PAO1

For the transcriptome analysis, PAO1 was grown on LB medium supplemented with the appropriate antibiotics. Cells were grown for 5 h in 100 ml flasks at 28°C with 200 rpm. For each sample point, two independent cultures were harvested after 5 hours of growth, immediately chilled on ice and stored at −70°C prior to mRNA extraction. From these samples, two separate cDNA libraries were generated from *P. aeruginosa* PAO1 harboring the pBBR1MCS-5::*bpiB09* and two libraries from the PAO1 control strain. Total RNA was isolated with the RNeasy Mini Kit (Qiagen) following the instructors manual resulting in 3 µg/µl in average. The rRNA was removed using the RiboMinus™ kit (Invitrogen). The obtained mRNA was randomly transcribed into single stranded cDNA with QuantiTect® Reverse Transcription Kit (Qiagen). Second strand synthesis was performed using the enzymes of the SuperScript™ Double-Stranded cDNA Synthesis Kit (Invitrogen) and following the instructions for the second strand synthesis. Double stranded cDNA samples were purified with SureClean solution (Bioline) and the resulting precipitate was resolved in 16 µl 50 mM Tris/HCL buffer. Starting from rRNA removal the whole procedure was performed in four attempts for each sample and samples were pooled. Concentration with an average of 6 ng/µl was determined using the NanoDrop1000 instrument (Peqlab Biotechnologie). The resulting ds cDNA was used to create 454-shotgun libraries following the GS Rapid library protocol (Roche) without any nebulization step. The four 454 cDNA libraries were sequenced with the Genome Sequencer FLX (Roche, Mannheim, Germany) using the Titanium chemistry. Sequencing was performed by the Göttingen Genomics Laboratory. One medium lane of a Titanium picotiter plate was used for sequencing of each sample. In total 567'003 reads were achieved for the four cDNA libraries. Total reads of all samples were mapped on the reference genome sequence of *P. aeruginosa* PAO1 and were evaluated with respect to the hits per gene, proportionately to the gene length.

### *C. elegans* paralysis assay

Paralysis assays were done as described in [40] with some modifications: PAO1 strains were grown on brain heart infusion (BHI) agar plates (Roth X915.1), supplemented with 50 µg/ml gentamycin to maintain plasmids. A single colony was picked and inoculated in BHI broth (Roth X916.1) at 37°C for 24 h. The culture was diluted 1∶100 into fresh BHI broth, 350 µl of the diluted culture were spread onto 100-mm BHI agar plates, and incubated at 37°C for 48 h. The *C. elegans* N2 strain was maintained on nematode growth medium (NGM) at 20°C, fed with *E. coli* OP50, and synchronized as described in [54]. 30 one-day-old adult *C. elegans* were picked onto each of 8 replicate plates per PAO1 strain. The assay was done at room temperature and plates were sealed with parafilm. The paralysis of the nematodes was scored every hour and worms were considered paralyzed if they did not move after repeatedly tapping the plate against the stage of the dissection microscope. The variation in percentage of paralyzed worms was evaluated with a generalized linear model, using ordinal logistic regression analysis and including the following factors in the model: PA strain, time, the PA strain*time interaction and also replicate nested in PA strain. In all cases, the specified model provided a better fit to the data than a minimal model (P<0.001) and it was not significantly worse than a saturated model (P>0.99). The analysis was performed with JMP version 8.0 (SAS Institute Inc. 2008). The graph was produced with SigmaPlot version 11.0 (Systat Software Inc. 2008).

### Crystallization

Purified BpiB09 was concentrated using a Vivaspin column (molecular-weight cutoff 10 kDa) to 10 mg/ml. Freshly prepared NADP^+^ (Karl Roth GmbH, Karlsruhe, Germany) was added to the sample immediately prior to crystallization to a final concentration of 1 mM (about 3-fold excess). Initial crystallization trials were carried out with five different 96 well screens from Qiagen (Classic I & II, PACT, AmSO_4_, PhClearII) at the EMBL Hamburg high-throughput crystallization facility [55]. All initial screens were performed at 292 K in Innovadyne plates using the sitting-drop vapor-diffusion method. 200 nL protein solution mixed with 200 nL reservoir solution was equilibrated against 50 µl reservoir solution. Initial crystallization experiments resulted in medium sized crystals under two conditions: 0.1 M citric acid pH 4.0, 1.6 M ammonium sulfate and 0.1 M tri sodium citrate pH 5.6, 2.5 M hexandiol, respectively. Only crystals grown from citric acid and ammonium sulfate diffracted x-ray radiation. They were subsequently optimized with customized screens using the hanging-drop method. Best diffraction was obtained with crystals grown from 0.1 M citric acid pH 4.5 and 1.5 M LiSO_4_. Equal volumes (1 µl) of protein solution (10 mg/ml) and reservoir solution were mixed and equilibrated against 1 ml of the reservoir solution at 292 K. Crystals appeared after 2–4 days and grew to dimensions of up to 300×300×200 µm^3^.

### Crystal Data Collection and Processing

For cryoprotection, a crystal of BpiB09 was briefly (2–5 s) immersed in mother liquor supplemented with 24% (v/v) ethyleneglycol and flash-frozen in liquid nitrogen. A complete X-ray diffraction data set was collected at beamline X13 at EMBL Hamburg/DESY on a MARCCD detector (165 mm). A total of 170 frames were collected with a rotation range of 0.4° based on the prediction of BEST [56]. The data were indexed and integrated using *XDS* [57] and scaled with *SCALA* (Collaborative Computational Project, Number 4, 1994). Intensities were converted to structure-factor amplitudes using the program *TRUNCATE* ([58]; Collaborative Computational Project, Number 4, 1994). Table 2 summarizes the data-collection and processing statistics.

### Structure Solution and Refinement

Phasing was accomplished by molecular replacement using the online molecular-replacement pipeline *BALBES* from the York Structural Biology Laboratory (http://www.ysbl.york.ac.uk/YSBLPrograms/index.jsp) [59]. The program selected the structure of a putative oxidoreductase from *Thermus thermophilus* as a search model (PDB entry 2ehd). 4 molecules were found in the asymmetric unit, which corresponds to a high solvent content of 76%. The structure was refined using *REFMAC5* [60] and Phenix 1.7 [61] alternating with manual rebuilding in *Coot* [62]. After few rounds of refinement unambiguous electron density remained at the expected site of NADP^+^ binding. Only after the R_free_ factor had dropped to below 24% gradual addition of the NADP^+^ cofactor into the model began. While there was continuous density for adenosine and the phosphoryl groups at the 2′ and 5′ hydroxyl side chains of the ribose, the density for the remaining phosphoryl-, ribose- and nicotinamide moiety was too weak for unambiguous tracing. Figures were prepared using PyMOL [63]. The atomic coordinates of BpiB09 have been deposited in the Protein Data Bank under the accession code 3RKR).

## Supporting Information

Figure S1 **Biofilm formation and swarming of** ***P. aeruginosa*** **carrying an empty vector pBBR1MCS-5 or pBBR1MCS-5::** ***celA*** **.** A) Biofilm formation of the two control strains. Both strains grew to a thickness of 30–40 µm after 72 h. B) Swarming of the two control strains. Both strains swarmed over the whole plate after 24 h.
(PDF)

Figure S2 **Analytical Ultracentrifugation of recombinant BpiB09 and di- and tertramerization mutants.** A) Analytical Ultracentrifugation of BpiB09. The elution peak between 105 and 110 kDa corresponds to the tetrameric mass of 109.6 kDa. B) Analytical Ultracentrifugation of c-terminally truncated BpiB09 (c-terminus is missing starting at F227). The elution peak corresponds to the monomeric mass of 27.4 kDa. C) Analytical Ultracentrifugation of BpiB09 with mutations G162Y and D109K. The elution peak corresponds to the monomeric mass.
(PDF)

Figure S3 **Phylogenetic analysis of BpiB09 and 39 closely related SDRs identified in the NCBI GenBank.** The phylogenetic tree was constructed using the T-Coffee and PhyML software in neighbor-joining mode and visualized using seaview program version 4.2.6. Amino acid sequences used to construct the phylogenetic tree were taken from GenBank, accession numbers follow the bacteria names. The asterisk indicates the Protein BpiB09. Bootstrap values, each expressed as a percentage of 100 replications, are given at branching points.
(PDF)

Figure S4 **HPLC-MS-MS.** A) MS-MS analysis of 3-oxo-C_12_-HSL incubated with BpiB09 detecting mass of 298.1 (blue line), the mass of 3-hydroxy-HSL, showing a clear peak at t = 14.0 min (upper panel). Lower panel: Mass spectrum recorded at t = 14.0 min, masses 112.0 and 141.9 correlate to fragments of 3-hydroxy-C_12_-HSL. B) MS-MS analysis of 3-oxo-C_12_-HSL incubated with BpiB09 detecting mass of 296.1 (red line), the mass of 3-oxo-C_12_-HSL, showing a peak at t = 14.5 min (upper panel). Lower panel: Mass spectrum recorded at t = 14.5 min, mass 171.0 correlates to a fragment of 3-oxo-C_12_-HSL. C) MS-MS analysis of 3-oxo-C_12_-HSL incubated with control eluate showing extracted mass of 298.1, the mass of 3-hydroxy-HSL (upper panel). Lower panel: Mass spectrum recorded at t = 14.0 min, masses 112.0 and 141.9 correlate to fragments of 3-hydroxy-C_12_-HSL at an intensity >10^2^ times lower than shown in A). D) MS-MS analysis of 3-oxo-C_12_-HSL incubated with control eluate showing extracted mass of 296.1, the mass of 3-oxo-C_12_-HSL (upper panel). Lower panel: Mass spectrum recorded at t = 14.5 min, mass 171.0 correlates to a fragment of 3-oxo-C_12_-HSL; the intensity is in the same range as B).
(PDF)

Table S1 Bacterial strains and plasmids used in this study.
(PDF)

Table S2 ≥10-fold repressed genes/ORFs in PAO1 expressing bpiB09^a^.
(PDF)

Table S3 ≥10-fold induced genes/ORFs in PAO1 expressing bpiB09^a^.
(PDF)

Table S4 Primers used in this study.
(PDF)
