# Supplementary figures and images for: A Novel Metagenomic Short-Chain Dehydrogenase/Reductase Attenuates Pseudomonas aeruginosa Biofilm Formation and Virulence on Caenorhabditis elegans
Source: PLoS One. 2011 Oct 26;6(10):e26278. doi: 10.1371/journal.pone.0026278 (PMC3202535; doi:10.1371/journal.pone.0026278)

**Figure S1**

**A**

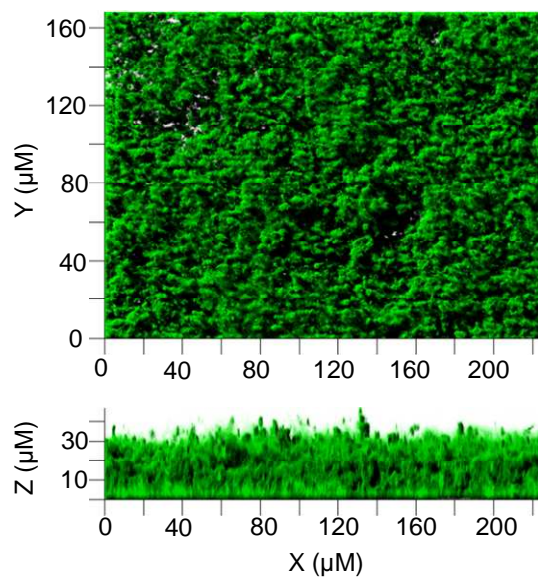

pBBR1MCS-5 (empty vector)

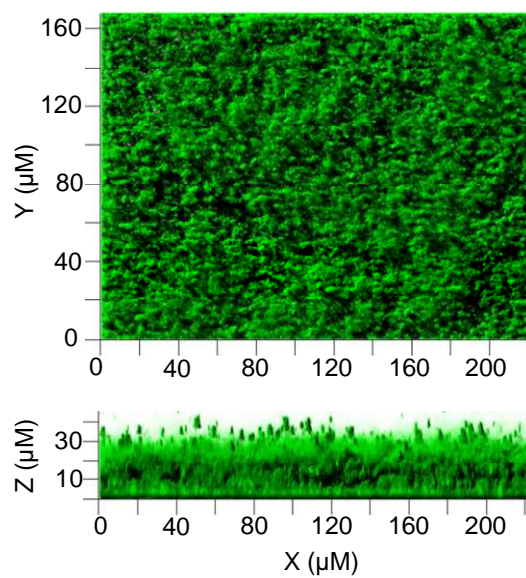

pBBR1MCS-5::ce/A

**B**

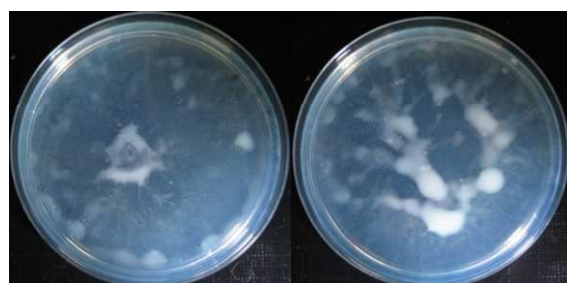

pBBR1MCS-5  
(empty vector)

pBBR1MCS-5::ce/A

Supplement: Figure S1 — Biofilm formation and swarming of P. aeruginosa carrying an empty vector pBBR1MCS-5 or pBBR1MCS-5:: celA . A) Biofilm formation of the two control strains. Both strains grew to a thickness of 30–40 µm after 72 h. B) Swarming of the two control strains. Both strains swarmed over the whole plate after 24 h. (PDF) [file pone.0026278.s001.pdf]

**Figure S2**

**A**

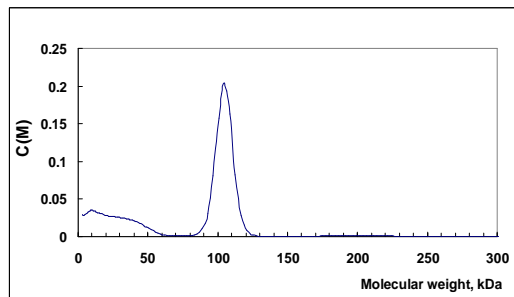

**B**

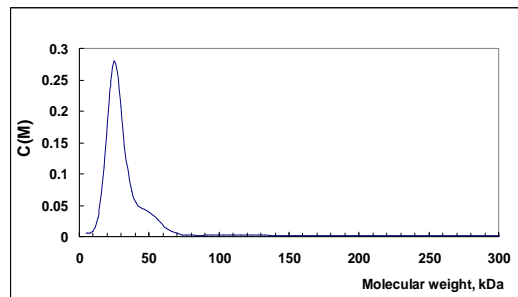

**C**

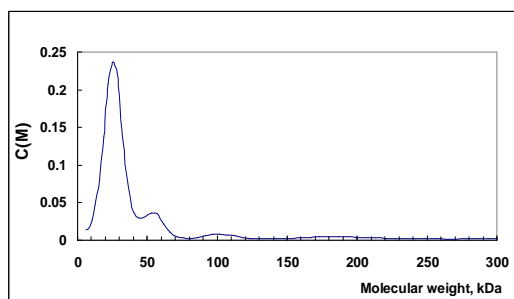

Supplement: Figure S2 — Analytical Ultracentrifugation of recombinant BpiB09 and di- and tertramerization mutants. A) Analytical Ultracentrifugation of BpiB09. The elution peak between 105 and 110 kDa corresponds to the tetrameric mass of 109.6 kDa. B) Analytical Ultracentrifugation of c-terminally truncated BpiB09 (c-terminus is missing starting at F227). The elution peak corresponds to the monomeric mass of 27.4 kDa. C) Analytical Ultracentrifugation of BpiB09 with mutations G162Y and D109K. The elution peak corresponds to the monomeric mass. (PDF) [file pone.0026278.s002.pdf]

Figure S3

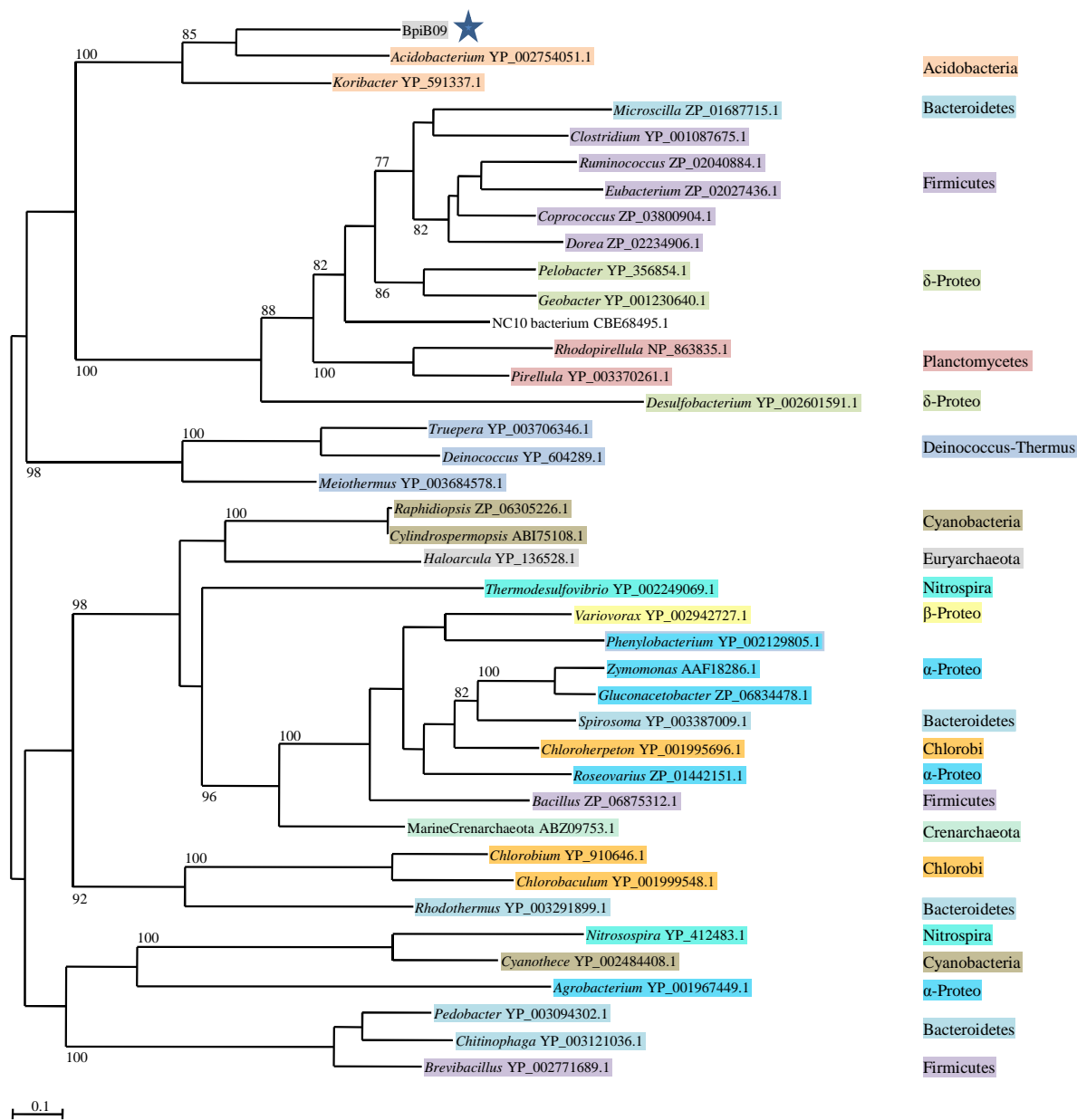

Supplement: Figure S3 — Phylogenetic analysis of BpiB09 and 39 closely related SDRs identified in the NCBI GenBank. The phylogenetic tree was constructed using the T-Coffee and PhyML software in neighbor-joining mode and visualized using seaview program version 4.2.6. Amino acid sequences used to construct the phylogenetic tree were taken from GenBank, accession numbers follow the bacteria names. The asterisk indicates the Protein BpiB09. Bootstrap values, each expressed as a percentage of 100 replications, are given at branching points. (PDF) [file pone.0026278.s003.pdf]

**Figure S4**

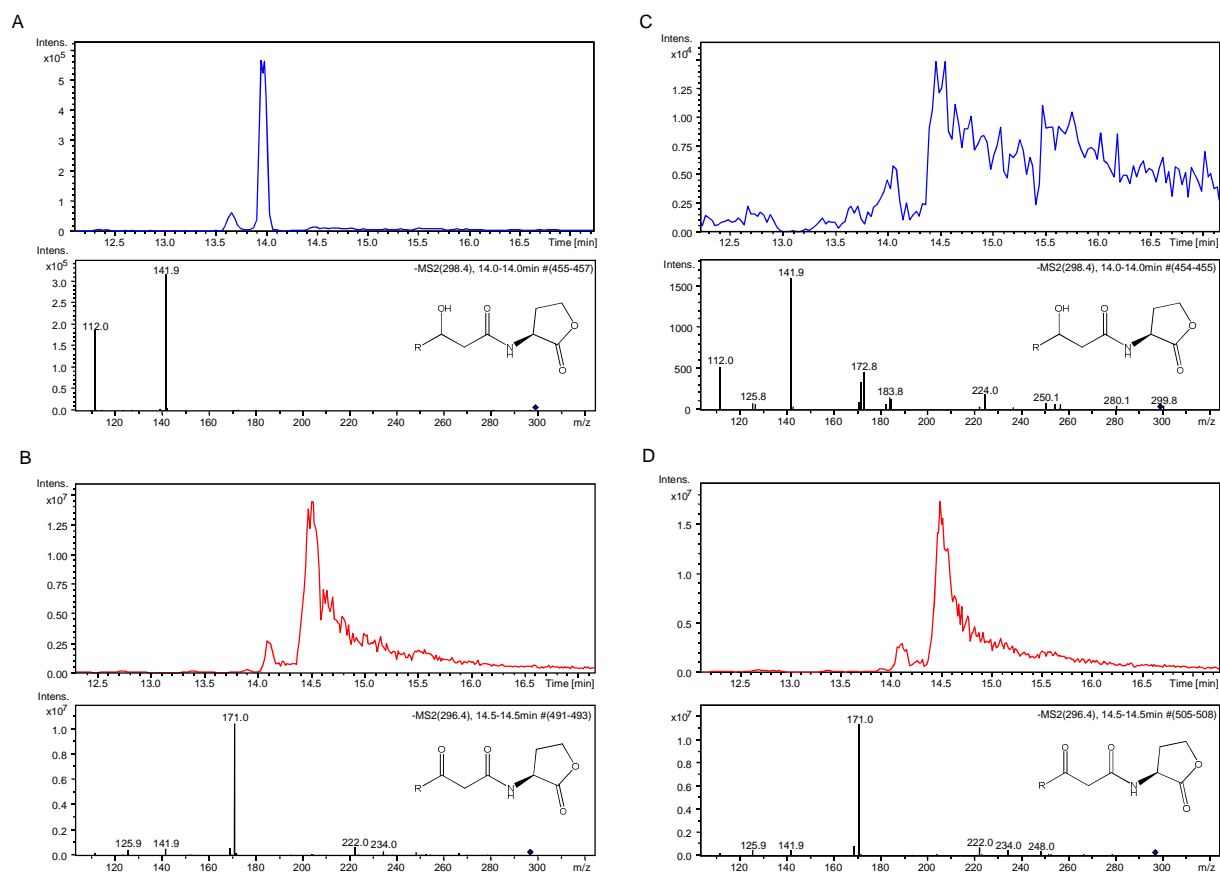

Supplement: Figure S4 — HPLC-MS-MS. A) MS-MS analysis of 3-oxo-C12-HSL incubated with BpiB09 detecting mass of 298.1 (blue line), the mass of 3-hydroxy-HSL, showing a clear peak at t = 14.0 min (upper panel). Lower panel: Mass spectrum recorded at t = 14.0 min, masses 112.0 and 141.9 correlate to fragments of 3-hydroxy-C12-HSL. B) MS-MS analysis of 3-oxo-C12-HSL incubated with BpiB09 detecting mass of 296.1 (red line), the mass of 3-oxo-C12-HSL, showing a peak at t = 14.5 min (upper panel). Lower panel: Mass spectrum recorded at t = 14.5 min, mass 171.0 correlates to a fragment of 3-oxo-C12-HSL. C) MS-MS analysis of 3-oxo-C12-HSL incubated with control eluate showing extracted mass of 298.1, the mass of 3-hydroxy-HSL (upper panel). Lower panel: Mass spectrum recorded at t = 14.0 min, masses 112.0 and 141.9 correlate to fragments of 3-hydroxy-C12-HSL at an intensity >102 times lower than shown in A). D) MS-MS analysis of 3-oxo-C12-HSL incubated with control eluate showing extracted mass of 296.1, the mass of 3-oxo-C12-HSL (upper panel). Lower panel: Mass spectrum recorded at t = 14.5 min, mass 171.0 correlates to a fragment of 3-oxo-C12-HSL; the intensity is in the same range as B). (PDF) [file pone.0026278.s004.pdf]
