# Supplementary material for: A Novel Metagenomic Short-Chain Dehydrogenase/Reductase Attenuates Pseudomonas aeruginosa Biofilm Formation and Virulence on Caenorhabditis elegans
Source: PLoS One. 2011 Oct 26;6(10):e26278. doi: 10.1371/journal.pone.0026278 (PMC3202535; doi:10.1371/journal.pone.0026278)
Supplement: Table S1 — Bacterial strains and plasmids used in this study. (PDF) [file pone.0026278.s005.pdf]

TABLE S1. Bacterial strains and plasmids used in this study.

Strain/ Plasmid	Properties	Reference
**Strains**		
E. coli DH5α	Cloning strain, *rec*A1, Δ*lac*Z	Gibco BRL now Invitrogen
E.coli BL21 (DE3)	Expression strain	[1]
P. aeruginosa PAO1	Parent strain	[2]
C. violaceum (DSM Nr. 30191)	Parent strain	DSMZ, Braunschweig
A. tumefaciens NTL4 (pCF218)(pCF372)	Reporter strain for autoinducer molecules, *traI::lacZ*, km^R, carrying pCF218 & pCF372	[3-5]
A. tumefaciens KYC6	AHL overproducing transposon mutant	[3]
**Plasmids**		
pBio5-pBKCMV	Metagenome-derived clone encoding *bpiB09* gene in pBK-CMV, km^R	This work, GenBank# EF530730.1,
pBluescript SK+	Cloning vector, amp^R	Fermentas/Thermo Scientific, St. Leon-Rot, Germany
pET-19a	N-terminal 10-his cloning vector, amp^R	Novagen, Karlsruhe, Germany
pET-21a	C-terminal 6-his cloning vector, amp^R	
pBBR1MCS-5	Broad host-range vector , km^R	[6]
pBK-CMV	Phagemid vector; km^R	Stratagene
pET19b::*bpiB09_dimer*	Dimerization mutants G162Y and D109K	This work
pET19b::*bpiB09_tetra*	Tetramerization mutant, G162Y and D109K and deletion of F227 to end	This work
pBBR1MCS-5::*bpiB09*	pBBR1MCS-5 containing *bpiB09*	This work
pBBR1MCS-5::*celA*	pBBR1MCS-5 containing 2 kb cellulase gene, experimental control	[7]
pBBR1MCS-5::*ACP_0942*	pBBR1MCS-5 containing *A. capsulatum* possible SDR gene ACP_0942	This work

1. Studier FW, Moffatt BA (1986) Use of bacteriophage T7 RNA polymerase to direct selective high-level expression of cloned genes. J Mol Biol 189: 113-130.
2. Holloway BW, Krishnapillai V, Morgan AF (1979) Chromosomal genetics of Pseudomonas. Microbiol Rev 43: 73-102.
3. Fuqua WC, Winans SC (1994) A LuxR-LuxI type regulatory system activates Agrobacterium Ti plasmid conjugal transfer in the presence of a plant tumor metabolite. J Bacteriol 176: 2796-2806.
4. Fuqua C, Winans SC (1996) Conserved cis-acting promoter elements are required for density-dependent transcription of Agrobacterium tumefaciens conjugal transfer genes. J Bacteriol 178: 435-440.
5. Luo ZQ, Clemente TE, Farrand SK (2001) Construction of a derivative of Agrobacterium tumefaciens C58 that does not mutate to tetracycline resistance. Mol Plant Microbe Interact 14: 98-103.
6. Kovach ME, Elzer PH, Hill DS, Robertson GT, Farris MA, et al. (1995) Four new derivatives of the broad-host-range cloning vector pBBR1MCS, carrying different antibiotic-resistance cassettes. Gene 166: 175-176.
7. Schipper C, Hornung C, Bijtenhoorn P, Quitschau M, Grond S, et al. (2009) Metagenome-derived clones encoding two novel lactonase family proteins involved in biofilm inhibition in Pseudomonas aeruginosa. Appl Environ Microbiol 75: 224-233.
