# Supplementary material for: A Novel Metagenomic Short-Chain Dehydrogenase/Reductase Attenuates Pseudomonas aeruginosa Biofilm Formation and Virulence on Caenorhabditis elegans
Source: PLoS One. 2011 Oct 26;6(10):e26278. doi: 10.1371/journal.pone.0026278 (PMC3202535; doi:10.1371/journal.pone.0026278)
Supplement: Table S2 — ≥10-fold repressed genes/ORFs in PAO1 expressing bpiB09a. (PDF) [file pone.0026278.s006.pdf]

Table S2. ≥10-fold repressed genes/ORFs in PAO1 expressing *bpiB09*[a]

ORF[b]	Gene	Description
PA0005	*lptA*	lysophosphatidic acid acyltransferase, LptA
PA0006		D,D-heptose 1,7-bisphosphate phosphatase
PA0007		hypothetical protein
PA0012		hypothetical protein
PA0031	*betC*	choline sulfatase
PA0033		hypothetical protein
PA0037	*trpI*	transcriptional regulator TrpI
PA0043		hypothetical protein
PA0049		hypothetical protein
PA0053		hypothetical protein
PA0056		transcriptional regulator
PA0057		hypothetical protein
PA0058		hypothetical protein
PA0060		hypothetical protein
PA0103		sulfate transporter
PA0104		hypothetical protein
PA0108	*coIII*	cytochrome c oxidase, subunit III
PA0109		hypothetical protein
PA0110		hypothetical protein
PA0114	*senC*	SenC
PA0117		short chain dehydrogenase
PA0122		hypothetical protein
PA0135		hypothetical protein
PA0136		ATP-binding component of ABC transporter
PA0137		permease of ABC transporter
PA0146		hypothetical protein
PA0149		sigma-70 factor, ECF subfamily
PA0150		transmembrane sensor
PA0151		TonB-dependent receptor
PA0153	*pcaH*	protocatechuate 3,4-dioxygenase, beta subunit
PA0155	*pcaR*	transcriptional regulator PcaR
PA0166		transporter
PA0173		chemotaxis-specific methylesterase
PA0178		two-component sensor
PA0179		two-component response regulator
PA0189		porin
PA0190		acid phosphatase
PA0191		transcriptional regulator
PA0192		TonB-dependent receptor
PA0194		hypothetical protein
PA0197	*tonB2*	TonB2
PA0209		triphosphoribosyl-dephospho-CoA synthase
PA0212	*mdcE*	malonate decarboxylase gamma subunit
PA0214		acyl transferase

ORF	Gene	Description
PA0220		amino acid APC family transporter
PA0234		hypothetical protein
PA0236		transcriptional regulator
PA0244		shikimate 5-dehydrogenase
PA0247	*pobA*	4-hydroxybenzoate 3-monooxygenase
PA0252		hypothetical protein
PA0256		hypothetical protein
PA0273		major facilitator superfamily (MFS) transporter
PA0274		hypothetical protein
PA0285		hypothetical protein
PA0288	*gpuA*	3-guanidinopropionase
PA0295		periplasmic polyamine binding protein
PA0307		hypothetical protein
PA0320		hypothetical protein
PA0323		binding protein component of ABC transporter
PA0324		permease of ABC transporter
PA0326		ATP-binding component of ABC transporter
PA0339		hypothetical protein
PA0345		hypothetical protein
PA0346		hypothetical protein
PA0355	*pfpI*	protease PfpI
PA0359		hypothetical protein
PA0364		oxidoreductase
PA0369		hypothetical protein
PA0371		hypothetical protein
PA0378		monofunctional biosynthetic peptidoglycan transglycosylase
PA0386		coproporphyrinogen III oxidase
PA0404		Holliday junction resolvase-like protein
PA0417	*chpE*	chemotaxis protein
PA0435		hypothetical protein
PA0439		dihydropyrimidine dehydrogenase
PA0440		oxidoreductase
PA0442		hypothetical protein
PA0452		stomatin-like protein
PA0455	*dbpA*	ATP-dependent RNA helicase DbpA
PA0466		hypothetical protein
PA0471		transmembrane sensor
PA0475		transcriptional regulator
PA0477		transcriptional regulator
PA0485		hypothetical protein
PA0486		serine/threonine protein kinase
PA0490		hypothetical protein
PA0491		transcriptional regulator
PA0492		hypothetical protein
PA0498		hypothetical protein
PA0539		hypothetical protein

ORF	Gene	Description
PA0544		hypothetical protein
PA0557		hypothetical protein
PA0560		hypothetical protein
PA0562		hydrolase
PA0565		hypothetical protein
PA0571		hypothetical protein
PA0572		hypothetical protein
PA0573		hypothetical protein
PA0575		hypothetical protein
PA0584	*cca*	multifunctional tRNA nucleotidyl transferase/2'3'-cyclic phosphodiesterase/2'nucleotidase/phosphatase
PA0599		hypothetical protein
PA0614		hypothetical protein
PA0618		bacteriophage protein
PA0671		hypothetical protein
PA0678		HxcU pseudopilin
PA0679		hypothetical protein
PA0681		HxcT pseudopilin
PA0683		type II secretion system protein
PA0684		type II secretion system protein
PA0692		hypothetical protein
PA0693	*exbB2*	transport protein ExbB2
PA0694	*exbD2*	transport protein ExbD
PA0695		hypothetical protein
PA0698		hypothetical protein
PA0703		major facilitator superfamily (MFS) transporter
PA0704		amidase
PA0707	*toxR*	transcriptional regulator ToxR
PA0708		transcriptional regulator
PA0725		hypothetical protein
PA0734		hypothetical protein
PA0739		transcriptional regulator
PA0740	*sdsA1*	SDS hydrolase SdsA1
PA0755	*opdH*	cis-aconitate porin OpdH
PA0756		two-component response regulator
PA0777		hypothetical protein
PA0781		hypothetical protein
PA0790		hypothetical protein
PA0808		hypothetical protein
PA0817		ring-cleaving dioxygenase
PA0821		hypothetical protein
PA0827		hypothetical protein
PA0841		hypothetical protein
PA0842		glycosyl transferase
PA0843	*plcR*	phospholipase accessory protein PlcR precursor
PA0844	*plcH*	hemolytic phospholipase C precursor

ORF	Gene	Description
PA0848		alkyl hydroperoxide reductase
PA0849	*trxB2*	thioredoxin reductase 2
PA0872	*phhA*	phenylalanine 4-monooxygenase
PA0877		transcriptional regulator
PA0882		hypothetical protein
PA0884		C4-dicarboxylate-binding periplasmic protein
PA0885		C4-dicarboxylate transporter
PA0907		hypothetical protein
PA0908		hypothetical protein
PA0914		hypothetical protein
PA0924		hypothetical protein
PA0926		hypothetical protein
PA0940		hypothetical protein
PA0977		hypothetical protein
PA0990		hypothetical protein
PA0996	*pqsA*	coenzyme A ligase
PA0997	*pqsB*	PqsB
PA0998	*pqsC*	PqsC
PA0999	*pqsD*	3-oxoacyl-(acyl carrier protein) synthase III
PA1000	*pqsE*	Quinolone signal response protein
PA1001	*phnA*	anthranilate synthase component I
PA1002	*phnB*	anthranilate synthase component II
PA1003	*mvfR*	Transcriptional regulator MvfR (pqsR)
PA1007		hypothetical protein
PA1019	*mucK*	cis,cis-muconate transporter MucK
PA1046		hypothetical protein
PA1067		transcriptional regulator
PA1079	*flgD*	flagellar basal body rod modification protein
PA1080	*flgE*	flagellar hook protein FlgE
PA1081	*flgF*	flagellar basal body rod protein FlgF
PA1088		hypothetical protein
PA1089		hypothetical protein
PA1092	*fliC*	flagellin type B
PA1093		hypothetical protein
PA1106		hypothetical protein
PA1120	*tpbB*	diguanylate cyclase TpbB
PA1130	*rhlC*	rhamnosyltransferase 2
PA1143		hypothetical protein
PA1154		hypothetical protein
PA1158		two-component sensor
PA1159		cold-shock protein
PA1172	*napC*	cytochrome c-type protein NapC
PA1177	*napE*	periplasmic nitrate reductase protein NapE
PA1182		transcriptional regulator
PA1185		glutathione S-transferase
PA1186		hypothetical protein

ORF	Gene	Description
PA1215		hypothetical protein
PA1220		hypothetical protein
PA1225		NAD(P)H dehydrogenase
PA1236		major facilitator superfamily (MFS) transporter
PA1239		hypothetical protein
PA1248	*aprF*	Alkaline protease secretion outer membrane protein
PA1250	*aprI*	alkaline proteinase inhibitor AprI
PA1251		chemotaxis transducer
PA1254		dihydrodipicolinate synthetase
PA1256		amino acid ABC transporter ATP binding protein
PA1257		amino acid ABC transporter membrane protein
PA1258		permease of ABC transporter
PA1259		hypothetical protein
PA1262		major facilitator superfamily (MFS) transporter
PA1265		hypothetical protein
PA1266		oxidoreductase
PA1270		hypothetical protein
PA1284		acyl-CoA dehydrogenase
PA1285		transcriptional regulator
PA1299		hypothetical protein
PA1300		sigma-70 factor, ECF subfamily
PA1302		heme utilization protein precursor
PA1310	*phnW*	2-aminoethylphosphonate--pyruvate transaminase
PA1313		major facilitator superfamily (MFS) transporter
PA1315		transcriptional regulator
PA1316		major facilitator superfamily (MFS) transporter
PA1319	*cyoC*	cytochrome o ubiquinol oxidase subunit III
PA1340		amino acid ABC transporter membrane protein
PA1341		amino acid ABC transporter membrane protein
PA1346		hypothetical protein
PA1348		hypothetical protein
PA1352		hypothetical protein
PA1367		hypothetical protein
PA1379		short chain dehydrogenase
PA1387		hypothetical protein
PA1388		hypothetical protein
PA1390		glycosyl transferase
PA1393	*cysC*	adenosine 5'-phosphosulfate (APS) kinase
PA1395		hypothetical protein
PA1396		two-component sensor
PA1407		hypothetical protein
PA1410		periplasmic spermidine/putrescine-binding protein
PA1418	*sodium*	solute symport protein
PA1423	*bdlA*	BdlA
PA1432	*lasI*	autoinducer synthesis protein LasI
PA1468		hypothetical protein

ORF	Gene	Description
PA1486		hypothetical protein
PA1495		hypothetical protein
PA1496		potassium channel
PA1500		oxidoreductase
PA1502	*gcl*	glyoxylate carboligase
PA1503		hypothetical protein
PA1513		hypothetical protein
PA1516		hypothetical protein
PA1525	*alkB2*	alkane-1-monooxygenase 2
PA1536		hypothetical protein
PA1538		flavin-containing monooxygenase
PA1558		hypothetical protein
PA1564		sulfur transfer protein SirA
PA1578		hypothetical protein
PA1608		chemotaxis transducer
PA1617		AMP-binding enzyme
PA1621		hydrolase
PA1625		hypothetical protein
PA1626		major facilitator superfamily (MFS) transporter
PA1635	*kdpC*	potassium-transporting ATPase subunit C
PA1637	*kdpE*	two-component response regulator KdpE
PA1641		hypothetical protein
PA1644		hypothetical protein
PA1646		chemotaxis transducer
PA1649		short chain dehydrogenase
PA1656		hypothetical protein
PA1660		hypothetical protein
PA1688		hypothetical protein
PA1696	*pscO*	translocation protein in type III secretion
PA1699		conserved hypothetical protein in type III secretion
PA1706	*pcrV*	type III secretion protein PcrV
PA1712	*exsB*	exoenzyme S synthesis protein B
PA1715	*pscB*	type III export apparatus protein
PA1721	*pscH*	type III export protein PscH
PA1725	*pscL*	type III secretion system protein
PA1728		hypothetical protein
PA1740		hypothetical protein
PA1741		hypothetical protein
PA1743		hypothetical protein
PA1745		hypothetical protein
PA1763		hypothetical protein
PA1765		hypothetical protein
PA1783	*nasA*	nitrate transporter
PA1784		hypothetical protein
PA1785		hypothetical protein
PA1827		short-chain dehydrogenase

ORF	Gene	Description
PA1839		ribosomal RNA large subunit methyltransferase N
PA1850		transcriptional regulator
PA1855		hypothetical protein
PA1860		hypothetical protein
PA1868	*xqhA*	secretion protein XqhA
PA1870		hypothetical protein
PA1872		hypothetical protein
PA1882		transporter
PA1887		hypothetical protein
PA1897		hypothetical protein
PA1898	*qscR*	quorum-sensing control repressor
PA1907		hypothetical protein
PA1915		hypothetical protein
PA1918		hypothetical protein
PA1922		TonB-dependent receptor
PA1924		hypothetical protein
PA1929		hypothetical protein
PA1932		hydroxylase molybdopterin-containing subunit
PA1935		hypothetical protein
PA1936		hypothetical protein
PA1954		hypothetical protein
PA1956		hypothetical protein
PA1958		transporter
PA1962	*azoR2*	azoreductase
PA1968		hypothetical protein
PA1972		hypothetical protein
PA1984	*exaC*	NAD+ dependent aldehyde dehydrogenase ExaC
PA1989	*pqqE*	pyrroloquinoline quinone biosynthesis protein PqqE
PA1994		hypothetical protein
PA1995		hypothetical protein
PA1999	*dhcA*	DhcA, dehydrocarnitine CoA transferase, subunit A
PA2000	*dhcB*	DhcB, dehydrocarnitine CoA transferase, subunit B
PA2005		transcriptional regulator
PA2009	*hmgA*	homogentisate 1,2-dioxygenase
PA2019		periplasmic multidrug efflux lipoprotein precursor
PA2034		hypothetical protein
PA2040		glutamine synthetase
PA2042		serine/threonine transporter SstT
PA2053	*cynT*	carbonate dehydratase
PA2056		transcriptional regulator
PA2060		permease of ABC transporter
PA2067		hydrolase
PA2069		carbamoyl transferase
PA2077		hypothetical protein
PA2085		ring-hydroxylating dioxygenase small subunit
PA2089		hypothetical protein

ORF	Gene	Description
PA2091		hypothetical protein
PA2093		RNA polymerase sigma factor
PA2100		transcriptional regulator
PA2109		hypothetical protein
PA2110		hypothetical protein
PA2111		hypothetical protein
PA2113	*opdO*	pyroglutatmate porin OpdO
PA2115		transcriptional regulator
PA2118	*ada*	O6-methylguanine-DNA methyltransferase
PA2121		transcriptional regulator
PA2130	*cupA3*	usher CupA3
PA2131	*cupA4*	fimbrial subunit CupA4
PA2133		hypothetical protein
PA2134		hypothetical protein
PA2137		hypothetical protein
PA2138		ATP-dependent DNA ligase
PA2141		hypothetical protein
PA2142		short-chain dehydrogenase
PA2144	*glgP*	glycogen phosphorylase
PA2148		hypothetical protein
PA2152		trehalose synthase
PA2154		hypothetical protein
PA2158		alcohol dehydrogenase (Zn-dependent)
PA2159		hypothetical protein
PA2162		maltooligosyl trehalose synthase
PA2164		glycosyl hydrolase
PA2168		hypothetical protein
PA2183		hypothetical protein
PA2184		hypothetical protein
PA2188		alcohol dehydrogenase (Zn-dependent)
PA2192		hypothetical protein
PA2193	*hcnA*	hydrogen cyanide synthase HcnA
PA2209		hypothetical protein
PA2213		porin
PA2216		hypothetical protein
PA2248	*bkdA2*	2-oxoisovalerate dehydrogenase (beta subunit)
PA2254	*pvcA*	paerucumarin biosynthesis protein PvcA
PA2255	*pvcB*	paerucumarin biosynthesis protein PvcB
PA2262		2-ketogluconate transporter
PA2263		2-hydroxyacid dehydrogenase
PA2278	*arsB*	ArsB protein
PA2284		hypothetical protein
PA2300	*chiC*	chitinase
PA2303	*ambD*	AmbD
PA2304	*ambC*	AmbC
PA2307		permease of ABC transporter

ORF	Gene	Description
PA2308		ATP-binding component of ABC transporter
PA2309		hypothetical protein
PA2312		transcriptional regulator
PA2319		transposase
PA2325		hypothetical protein
PA2336		hypothetical protein
PA2348		hypothetical protein
PA2350		ATP-binding component of ABC transporter
PA2352		glycerophosphoryl diester phosphodiesterase
PA2354		transcriptional regulator
PA2359		transcriptional regulator
PA2366		uricase PuuD
PA2368		hypothetical protein
PA2380		hypothetical protein
PA2383		transcriptional regulator
PA2389	*pvdR*	PvdR
PA2404		hypothetical protein
PA2405		hypothetical protein
PA2408		ATP-binding component of ABC transporter
PA2411		thioesterase
PA2412		hypothetical protein
PA2418		hypothetical protein
PA2425	*pvdG*	PvdG
PA2426	*pvdS*	extracytoplasmic-function sigma-70 factor
PA2428		hypothetical protein
PA2435		cation-transporting P-type ATPase
PA2438		hypothetical protein
PA2444	*glyA2*	serine hydroxymethyltransferase
PA2446	*gcvH2*	glycine cleavage system protein H
PA2452		hypothetical protein
PA2453		hypothetical protein
PA2460		hypothetical protein
PA2461		hypothetical protein
PA2465		hypothetical protein
PA2469		transcriptional regulator
PA2471		hypothetical protein
PA2472		major facilitator superfamily (MFS) transporter
PA2474		hypothetical protein
PA2475		cytochrome P450
PA2476	*dsbG*	disulfide isomerase/thiol-disulfide oxidase
PA2477	*thiol*	disulfide interchange protein
PA2479		two-component response regulator
PA2485		hypothetical protein
PA2490		hypothetical protein
PA2494	*mexF*	Resistance-Nodulation-Cell Division (RND) multidrug efflux transporter MexF
PA2495	*oprN*	Multidrug efflux outer membrane protein OprN precursor

ORF	Gene	Description
PA2496		hypothetical protein
PA2498		hypothetical protein
PA2507	*catA*	catechol 1,2-dioxygenase
PA2508	*catC*	muconolactone delta-isomerase
PA2517	*xylY*	toluate 1,2-dioxygenase beta subunit
PA2521	*czcB*	Resistance-Nodulation-Cell Division (RND) divalent metal cation efflux membrane fusion protein CzcB precursor
PA2522	*czcC*	outer membrane protein precursor CzcC
PA2544		hypothetical protein
PA2555		AMP-binding enzyme
PA2561		chemotaxis transducer
PA2577		transcriptional regulator
PA2580		hypothetical protein
PA2590		hypothetical protein
PA2591		transcriptional regulator VqsR
PA2592		periplasmic spermidine/putrescine-binding protein
PA2594		hypothetical protein
PA2610		hypothetical protein
PA2617	*aat*	leucyl/phenylalanyl-tRNA--protein transferase
PA2618		arginyl-tRNA-protein transferase
PA2625		hypothetical protein
PA2666		6-pyruvoyl tetrahydrobiopterin synthase
PA2668		hypothetical protein
PA2671		hypothetical protein
PA2672		type II secretion system protein
PA2673		type II secretion system protein
PA2699		hypothetical protein
PA2701		major facilitator superfamily (MFS) transporter
PA2704		transcriptional regulator
PA2711		periplasmic spermidine/putrescine-binding protein
PA2714		molybdopterin oxidoreductase
PA2719		hypothetical protein
PA2721		hypothetical protein
PA2722		hypothetical protein
PA2745		hydrolase
PA2747		hypothetical protein
PA2767		enoyl-CoA hydratase/isomerase
PA2778		hypothetical protein
PA2782		hypothetical protein
PA2783		hypothetical protein
PA2788		chemotaxis transducer
PA2791		hypothetical protein
PA2795		tRNA-dihydrouridine synthase A
PA2804		hypothetical protein
PA2807		hypothetical protein
PA2809	*copR*	two-component response regulator, CopR

ORF	Gene	Description
PA2816		hypothetical protein
PA2832	*tpm*	thiopurine S-methyltransferase
PA2833		hypothetical protein
PA2834		transcriptional regulator
PA2835		major facilitator superfamily (MFS) transporter
PA2868		hypothetical protein
PA2869		hypothetical protein
PA2871		hypothetical protein
PA2872		hypothetical protein
PA2875		hypothetical protein
PA2890	*atuE*	isohexenylglutaconyl-CoA hydratase
PA2892	*atuG*	short chain dehydrogenase
PA2905	*cobH*	precorrin-8X methylmutase
PA2909		cobalt-precorrin-6x reductase
PA2911		TonB-dependent receptor
PA2912		ATP-binding component of ABC transporter
PA2916		hypothetical protein
PA2918		short chain dehydrogenase
PA2923	*hisJ*	periplasmic histidine-binding protein HisJ
PA2924	*hisQ*	histidine transport system permease HisQ
PA2934	*cif*	CFTR inhibitory factor, Cif
PA2936		hypothetical protein
PA2938		transporter
PA2940		acyl-CoA thiolase
PA2985		hypothetical protein
PA2989		hypothetical protein
PA3023		lipid kinase
PA3035		glutathione S-transferase
PA3044		two-component sensor
PA3061	*pelD*	PelD
PA3063	*pelB*	PelB
PA3078		two-component sensor
PA3119		hypothetical protein
PA3130		hypothetical protein
PA3132		hydrolase
PA3133		transcriptional regulator
PA3140		hypothetical protein
PA3174		transcriptional regulator
PA3175		formimidoylglutamase
PA3176	*gltS*	glutamate/sodium ion symporter, GltS
PA3186	*oprB*	Glucose/carbohydrate outer membrane porin OprB precursor
PA3187		ATP-binding component of ABC transporter
PA3188		permease of ABC sugar transporter
PA3209		hypothetical protein
PA3216		hypothetical protein
PA3218		hypothetical protein

ORF	Gene	Description
PA3219		hypothetical protein
PA3223	*azoR3*	AzoR3, azoreductase 3
PA3224		hypothetical protein
PA3230		hypothetical protein
PA3240		hypothetical protein
PA3248		hypothetical protein
PA3251		hypothetical protein
PA3259		hypothetical protein
PA3273		hypothetical protein
PA3274		hypothetical protein
PA3279	*oprP*	Phosphate-specific outer membrane porin OprP precursor
PA3282		hypothetical protein
PA3293		hypothetical protein
PA3307		hypothetical protein
PA3318		hypothetical protein
PA3319	*plcN*	non-hemolytic phospholipase C precursor
PA3325		hypothetical protein
PA3328		FAD-dependent monooxygenase
PA3329		hypothetical protein
PA3331		cytochrome P450
PA3332		hypothetical protein
PA3333	*fabH2*	3-oxoacyl-(acyl carrier protein) synthase III
PA3334		acyl carrier protein
PA3335		hypothetical protein
PA3348		chemotaxis protein methyltransferase
PA3349		chemotaxis protein
PA3355		hypothetical protein
PA3358		hypothetical protein
PA3359		hypothetical protein
PA3370		hypothetical protein
PA3371		hypothetical protein
PA3376		phosphonate C-P lyase system protein PhnK
PA3379		carbon-phosphorus lyase complex subunit
PA3380		hypothetical protein
PA3381		transcriptional regulator
PA3383		binding protein component of ABC phosphonate transporter
PA3388		hypothetical protein
PA3394	*nosF*	NosF protein
PA3395	*nosY*	NosY protein
PA3406	*hasD*	transport protein HasD
PA3410		sigma-70 factor, ECF subfamily
PA3413		hypothetical protein
PA3417		pyruvate dehydrogenase E1 component, alpha subunit
PA3421		hypothetical protein
PA3422		hypothetical protein
PA3425		hypothetical protein

ORF	Gene	Description
PA3429		epoxide hydrolase
PA3436		hypothetical protein
PA3444		alkanesulfonate monooxygenase
PA3445		hypothetical protein
PA3447		ATP-binding component of ABC transporter
PA3449		hypothetical protein
PA3457		hypothetical protein
PA3463		hypothetical protein
PA3472		hypothetical protein
PA3476	*rhlI*	autoinducer synthesis protein RhlI
PA3478	*rhlB*	rhamnosyltransferase chain B
PA3479	*rhlA*	rhamnosyltransferase chain A
PA3486		hypothetical protein
PA3493		hypothetical protein
PA3497		hypothetical protein
PA3498		oxidoreductase
PA3505		L-aspartate dehydrogenase
PA3506		hypothetical protein
PA3509		hydrolase
PA3510		hypothetical protein
PA3511		short chain dehydrogenase
PA3515		hypothetical protein
PA3519		hypothetical protein
PA3523		Resistance-Nodulation-Cell Division (RND) efflux membrane fusion protein precursor
PA3530		hypothetical protein
PA3537	*argF*	ornithine carbamoyltransferase
PA3541	*alg8*	alginate biosynthesis protein Alg8
PA3544	*algE*	Alginate production outer membrane protein AlgE precursor
PA3546	*algX*	alginate biosynthesis protein AlgX
PA3547	*algL*	poly(beta-D-mannuronate) lyase
PA3551	*algA*	phosphomannose isomerase / guanosine 5'-diphospho-D-mannose pyrophosphorylase
PA3564		hypothetical protein
PA3566		hypothetical protein
PA3569	*mmsB*	3-hydroxyisobutyrate dehydrogenase
PA3588		porin
PA3593		acyl-CoA dehydrogenase
PA3595		major facilitator superfamily (MFS) transporter
PA3597		amino acid permease
PA3598		hypothetical protein
PA3611		hypothetical protein
PA3630		transcriptional regulator
PA3678		transcriptional regulator
PA3684		hypothetical protein
PA3713	*spdH*	spermidine dehydrogenase, SpdH
PA3722		hypothetical protein

ORF	Gene	Description
PA3724	*lasB*	elastase LasB
PA3734		hypothetical protein
PA3747		hypothetical protein
PA3750		hypothetical protein
PA3758		N-acetylglucosamine-6-phosphate deacetylase
PA3762		hypothetical protein
PA3765		hypothetical protein
PA3772		hypothetical protein
PA3773		hypothetical protein
PA3775		hypothetical protein
PA3818		extragenic suppressor protein SuhB
PA3825		hypothetical protein
PA3829		hypothetical protein
PA3842		chaperone
PA3844		hypothetical protein
PA3845		transcriptional regulator
PA3854		GIY-YIG nuclease superfamily protein
PA3855		hypothetical protein
PA3867		DNA invertase
PA3885	*tpbA*	protein tyrosine phosphatase TpbA
PA3888		permease of ABC transporter
PA3905		hypothetical protein
PA3906		hypothetical protein
PA3907		hypothetical protein
PA3908		hypothetical protein
PA3909	*eddB*	Extracelullar DNA degradation protein, EddB
PA3924		long-chain-fatty-acid--CoA ligase
PA3928		hypothetical protein
PA3931		hypothetical protein
PA3935	*tauD*	taurine dioxygenase
PA3937		ATP-binding component of ABC taurine transporter
PA3943		hypothetical protein
PA3947	*rocR*	RocR
PA3953		hypothetical protein
PA3954		hypothetical protein
PA3958		hypothetical protein
PA3959		hypothetical protein
PA3962		hypothetical protein
PA3963		transporter
PA3966		hypothetical protein
PA3967		hypothetical protein
PA3976	*thiE*	thiamine-phosphate pyrophosphorylase
PA3986		hypothetical protein
PA3991		hypothetical protein
PA4018		hypothetical protein
PA4026		acetyltransferase

ORF	Gene	Description
PA4033		hypothetical protein
PA4034	*aqpZ*	aquaporin Z
PA4037		ATP-binding component of ABC transporter
PA4040		hypothetical protein
PA4041		hypothetical protein
PA4046		hypothetical protein
PA4064		ATP-binding component of ABC transporter
PA4070		DNA-binding transcriptional activator FeaR
PA4071		hypothetical protein
PA4076		hypothetical protein
PA4082	*cupB5*	adhesive protein CupB5
PA4099		hypothetical protein
PA4104		hypothetical protein
PA4106		hypothetical protein
PA4113		sugar efflux transporter
PA4119	*aph*	aminoglycoside 3'-phosphotransferase type IIb
PA4125	*hpcD*	5-carboxymethyl-2-hydroxymuconate isomerase
PA4136		major facilitator superfamily (MFS) transporter
PA4139		hypothetical protein
PA4144		outer membrane protein precursor
PA4160	*fepD*	ferric enterobactin transport protein FepD
PA4164		hypothetical protein
PA4169		hypothetical protein
PA4172		nuclease
PA4185		transcriptional regulator
PA4188		hypothetical protein
PA4189		aldehyde dehydrogenase
PA4190	*pqsL*	monooxygenase
PA4191		iron/ascorbate oxidoreductase
PA4193		permease of ABC transporter
PA4194		permease of ABC transporter
PA4195		binding protein component of ABC transporter
PA4208	*opmD*	outer membrane protein precursor
PA4209	*phzM*	phenazine-specific methyltransferase
PA4211	*phzB1*	phenazine biosynthesis protein
PA4219		hypothetical protein
PA4220		hypothetical protein
PA4221	*fptA*	Fe(III)-pyochelin outer membrane receptor precursor
PA4224	*pchG*	pyochelin biosynthetic protein PchG
PA4225	*pchF*	pyochelin synthetase
PA4227	*pchR*	transcriptional regulator PchR
PA4229	*pchC*	pyochelin biosynthetic protein PchC
PA4290		chemotaxis transducer
PA4291		hypothetical protein
PA4293	*pprA*	two-component sensor PprA
PA4301	*tadB*	TadB

ORF	Gene	Description
PA4303	*tadZ*	TadZ
PA4304	*rcpA*	RcpA
PA4326		hypothetical protein
PA4341		transcriptional regulator
PA4344		hydrolase
PA4362		hypothetical protein
PA4369		hypothetical protein
PA4396		two-component response regulator
PA4397	*panE*	2-dehydropantoate 2-reductase
PA4422		hypothetical protein
PA4436		transcriptional regulator
PA4437		hypothetical protein
PA4469		hypothetical protein
PA4470	*fumC1*	fumarate hydratase
PA4481	*mreB*	rod shape-determining protein MreB
PA4504		permease of ABC transporter
PA4505		ATP-binding component of ABC transporter
PA4507		hypothetical protein
PA4509		hypothetical protein
PA4510		hypothetical protein
PA4541		hypothetical protein
PA4543		hypothetical protein
PA4544	*rluD*	pseudouridine synthase
PA4563	*rpsT*	30S ribosomal protein S20
PA4570		hypothetical protein
PA4589		outer membrane protein precursor
PA4590	*pra*	protein activator
PA4591		hypothetical protein
PA4601	*morA*	motility regulator
PA4623		hypothetical protein
PA4629		hypothetical protein
PA4633		chemotaxis transducer
PA4651		pili assembly chaperone
PA4679		hypothetical protein
PA4681		hypothetical protein
PA4683		hypothetical protein
PA4692		sulfite oxidase subunit YedY
PA4697		hypothetical protein
PA4702		hypothetical protein
PA4703		hypothetical protein
PA4712		hypothetical protein
PA4719		transporter
PA4738		hypothetical protein
PA4786		3-ketoacyl-(acyl-carrier-protein) reductase
PA4788		hypothetical protein
PA4789		hypothetical protein

ORF	Gene	Description
PA4791		hypothetical protein
PA4802		hypothetical protein
PA4806		transcriptional regulator
PA4817		hypothetical protein
PA4820		hypothetical protein
PA4822		hypothetical protein
PA4825	*mgtA*	Mg(2+) transport ATPase, P-type 2
PA4834		hypothetical protein
PA4844		chemotaxis transducer
PA4849		hypothetical protein
PA4851		hypothetical protein
PA4858		hypothetical protein
PA4859		permease of ABC transporter
PA4861		ATP-binding component of ABC transporter
PA4862		ATP-binding component of ABC transporter
PA4864	*ureD*	urease accessory protein
PA4865	*ureA*	urease subunit gamma
PA4871		hypothetical protein
PA4875		hypothetical protein
PA4880		bacterioferritin
PA4891	*ureE*	urease accessory protein UreE
PA4895		transmembrane sensor
PA4911		permease of ABC branched-chain amino acid transporter
PA4914		transcriptional regulator
PA4925		hypothetical protein
PA4927		hypothetical protein
PA4978		hypothetical protein
PA4979		acyl-CoA dehydrogenase
PA4985		hypothetical protein
PA4986		oxidoreductase
PA4993		hypothetical protein
PA5024		hypothetical protein
PA5033		hypothetical protein
PA5050	*priA*	primosome assembly protein PriA
PA5057	*phaD*	poly(3-hydroxyalkanoic acid) depolymerase
PA5058	*phaC2*	poly(3-hydroxyalkanoic acid) synthase 2
PA5059		transcriptional regulator
PA5072		chemotaxis transducer
PA5095		permease of ABC transporter
PA5101		hypothetical protein
PA5123		hypothetical protein
PA5127		rRNA methylase
PA5144		hypothetical protein
PA5154		permease of ABC transporter
PA5157		transcriptional regulator
PA5167		c4-dicarboxylate-binding protein

ORF	Gene	Description
PA5168		dicarboxylate transporter
PA5180		hypothetical protein
PA5191		hypothetical protein
PA5221		2-octaprenyl-3-methyl-6-methoxy-1,4-benzoquinol hydroxylase
PA5246		hypothetical protein
PA5247		hypothetical protein
PA5249		hypothetical protein
PA5280	*sss*	site-specific tyrosine recombinase XerC
PA5284		hypothetical protein
PA5293		transcriptional regulator
PA5294		multidrug efflux protein NorA
PA5311		major facilitator superfamily (MFS) transporter
PA5314		hypothetical protein
PA5348		DNA-binding protein
PA5353	*glcF*	glycolate oxidase iron-sulfur subunit
PA5370		major facilitator superfamily (MFS) transporter
PA5375	*betT1*	choline transporter BetT
PA5383		hypothetical protein
PA5384		lipolytic enzyme
PA5385	*cdhB*	CdhB, Carnitine dehydrogenase-related protein B
PA5391		hypothetical protein
PA5400		electron transfer flavoprotein alpha subunit
PA5402		hypothetical protein
PA5418	*soxA*	sarcosine oxidase alpha subunit
PA5437		transcriptional regulator
PA5442		hypothetical protein
PA5444		hypothetical protein
PA5462		hypothetical protein
PA5463		hypothetical protein
PA5464		hypothetical protein
PA5465		hypothetical protein
PA5469		hypothetical protein
PA5471.1		leader peptide
PA5480		hypothetical protein
PA5482		hypothetical protein
PA5501	*znuB*	permease of ABC zinc transporter ZnuB
PA5506		hypothetical protein
PA5507		hypothetical protein
PA5513	*poxA*	hypothetical protein
PA5516	*pdxY*	pyridoxamine kinase
PA5519		hypothetical protein
PA5520		hypothetical protein
PA5534		hypothetical protein
PA5539		GTP cyclohydrolase
PA5541	*pyrQ*	dihydroorotase
PA5543		hypothetical protein

[a] *P. aeruginosa* PAO1 expressing *bpiB09* compared to a control-strain with empty vector, samplepoint 5h.
[b] Gene Number, Name and description are from the *Pseudomonas* genome project [33].
