# Supplementary material for: A Novel Metagenomic Short-Chain Dehydrogenase/Reductase Attenuates Pseudomonas aeruginosa Biofilm Formation and Virulence on Caenorhabditis elegans
Source: PLoS One. 2011 Oct 26;6(10):e26278. doi: 10.1371/journal.pone.0026278 (PMC3202535; doi:10.1371/journal.pone.0026278)
Supplement: Table S3 — ≥10-fold induced genes/ORFs in PAO1 expressing bpiB09a. (PDF) [file pone.0026278.s007.pdf]

Table S3. ≥10-fold induced genes/ORFs in PAO1 expressing *bpiB09*[a]

**ORF**[b]	**Gene**	**Description**
PA2222		hypothetical protein
PA2223		hypothetical protein
PA2224		hypothetical protein
PA2225		hypothetical protein
PA2226		hypothetical protein
PA2227	*vqsM*	AraC-type transcriptional regulator VqsM
PA2228		hypothetical protein
PA2694		thioredoxin

[a] *P. aeruginosa* PAO1 expressing *bpiB09* compared to a control-strain with empty vector, samplepoint 5h.
[b] Gene Number, Name and description are from the *Pseudomonas* genome project [8].
