# Supplementary material for: A Novel Metagenomic Short-Chain Dehydrogenase/Reductase Attenuates Pseudomonas aeruginosa Biofilm Formation and Virulence on Caenorhabditis elegans
Source: PLoS One. 2011 Oct 26;6(10):e26278. doi: 10.1371/journal.pone.0026278 (PMC3202535; doi:10.1371/journal.pone.0026278)
Supplement: Table S4 — Primers used in this study. (PDF) [file pone.0026278.s008.pdf]

TABLE S4. Primers used in this study.

ORF	Primer 5' – 3'
bpiB09_pBBR	for: CTAAGCTTACTGGGGAATGTTCCGTTGA<br>rev: GCAAGCTTGAAATAAGCACAGCTCTCGA
bpiB09_pet	for: GACATATGAGTTCCCTTTCCGGACA<br>rev: GCGGATCCCTTCTTCAGCGTAGGCCGCA
ACP_0942_pBBR	for: ATGAAGCTTTGGAACCTGGCCGGAAGAGG<br>rev: ATGGGATCCAGTCGCCGCTCGTCAGCATC
B09-D109K	for: ACCGGCGGAGTGGAAAGCGCTGATCGCGG<br>rev: TTCATCGTGTGCAGCGGCCCGCCGAACCA
B09- G162Y	for: CGGCTTCAAAATGGTACTTGAACGGGCTGA<br>rev: TATACGCCGCCCCATCCGCCACCGGATT
B09-Δ227-239	for: TAGCTCGAGGATCCGGCTGCTAACA<br>rev: AGACTGGTCCGCCTGCGTCGCCA
